# Single‐cell RNA‐Seq reveals a highly coordinated transcriptional program in mouse germ cells during primordial follicle formation

**DOI:** 10.1111/acel.13424

**Published:** 2021-06-26

**Authors:** Yuanlin He, Qiuzhen Chen, Juncheng Dai, Yiqiang Cui, Chi Zhang, Xidong Wen, Jiazhao Li, Yue Xiao, Xiaoxu Peng, Mingxi Liu, Bin Shen, Jiahao Sha, Zhibin Hu, Jing Li, Wenjie Shu

**Affiliations:** ^1^ State Key Laboratory of Reproductive Medicine Nanjing Medical University Nanjing China; ^2^ Department of Epidemiology and Biostatistics International Joint Research Center on Environment and Human Health Center for Global Health School of Public Health Nanjing Medical University Nanjing China; ^3^ Department of Biotechnology Beijing Institute of Radiation Medicine Beijing China; ^4^ Computer School University of South China Hengyang China

**Keywords:** cyst breakdown, oocyte, ovary, primordial follicle, single‐cell RNA‐seq

## Abstract

The assembly of primordial follicles in mammals represents one of the most critical processes in ovarian biology. It directly affects the number of oocytes available to a female throughout her reproductive life. Premature depletion of primordial follicles contributes to the ovarian pathology primary ovarian insufficiency (POI). To delineate the developmental trajectory and regulatory mechanisms of oocytes during the process, we performed RNA‐seq on single germ cells from newborn (P0.5) ovaries. Three cell clusters were classified which corresponded to three cell states (germ cell cyst, cyst breakdown, and follicle) in the newborn ovary. By Monocle analysis, a uniform trajectory of oocyte development was built with a series of genes showed dynamic changes along the pseudo‐timeline. Gene Ontology term enrichment revealed a significant decrease in meiosis‐related genes and a dramatic increase in oocyte‐specific genes which marked the transition from a germ cell to a functional oocyte. We then established a network of regulons by using single‐cell regulatory network inference and clustering (SCENIC) algorithm and identified possible candidate transcription factors that may maintain transcription programs during follicle formation. Following functional studies further revealed the differential regulation of the identified regulon *Id2* and its family member *Id1*, on the establishment of primordial follicle pool by using siRNA knockdown and genetic modified mouse models. In summary, our study systematically reconstructed molecular cascades in oocytes and identified a series of genes and molecular pathways in follicle formation and development.

## Graphical abstract

The assembly of primordial follicle determines the number of oocytes available to a female throughout her reproductive life. Premature depletion of primordial follicles contributes to the ovarian pathology primary ovarian insufficiency. We performed RNA‐seq on single germ cells from newborn mouse ovary and the data revealed dynamic gene expressions along with the transition of germ cells to a functional oocyte. Our study highlights the importance of transcriptional regulations on the process.

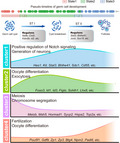

## 1 INTRODUCTION

In mammals, it is widely accepted that fixed numbers of primordial follicles are formed to provide a source of fertilizable oocytes in the reproductive lifespan. Along with the continuous and cyclic follicular development after puberty, the cohort of primordial follicles shrinks steadily until it is finally depleted, and a series of physiological changes known as menopause occurs (Rossetti et al., 2017). In humans, menopause occurs, on average, at 51 years of age (range 40–60 years); however, approximately 1%–2% of women worldwide suffer primary ovarian insufficiency (POI), a kind of ovarian dysfunction related to very early aging of the ovaries which is characterized by the amenorrhea before 40 years of age (Kirshenbaum & Orvieto, 2019). Inadequate follicles during the establishment of pool will cause POI and, in some cases, POI patients harbor mutations in genes responsible for the formation of primordial follicles and initial recruitment (Qin et al., 2014, 2015; Zhao et al., 2008). Thus, based on the particular relevance of the primordial follicle pool with some other processes of folliculogenesis, in‐depth studies of the follicular assembly will provide a better understanding of the genetic basis of POI.

In humans, primordial follicle formation begins during mid‐gestation, while in mice, it begins at E17.5 (17.5 dpc) and is completed within the first 3 days after birth. Before follicle formation, germ cells form germ‐line cysts by synchronous, incomplete mitotic divisions. The cysts then undergo a progressive loss of synchrony by partial fragmentation into smaller cysts and associate with other unrelated cysts to form germ‐line nests just prior to meiosis initiation at E14.5 (Cui et al., 2013). The subsequent steps include meiosis initiation, cyst breakdown (CBD), and follicle assembly, which occur in the ovary in a temporally and spatially asynchronous manner. In mice, meiosis initiates via an anterior‐to‐posterior pattern along the axis of the ovary; the onset of CBD and follicle assembly starts from the medullar region to the cortex. The primordial follicles formed in the medullar region are immediately activated and then depleted early after puberty, whereas the primordial follicles formed in the cortex remain dormant and are gradually activated throughout the entire course of the reproductive lifespan (Zheng et al., 2014). Another characteristic of follicle assembly is the loss of a large number of oocytes by programmed cell death (PCD), and only one‐third of oocytes remain to form primordial follicles. A recent study found that mouse germ cells receive organelles from neighboring cyst cells and build a balbiani body to become oocytes encapsulated in primordial follicles (Lei & Spradling, 2016). The results suggest the selection of oocytes that are destined to form follicles. However, the underlying mechanism remains unknown, and it is difficult to anticipate the fate of each cell during the process.

The importance of oocytes on ovarian determination has long been acknowledged. During oogenesis, the oocyte undergoes dynamic alterations in gene expression that are regulated by a set of germ cell‐specific transcription factors. These regulators include FIGLA, NOBOX, LHX8, SOHLH1, and SOHLH2; Mutations in *FIGLA* and *NOBOX* have also been reported in human POI patients (Rossetti et al., 2017). Ovaries from mutants of the above genes are devoid of follicles shortly after birth, exhibiting defects in primordial follicle formation. The interrelationships among these transcriptional regulators have been revealed by microarray analyses of related gene knockout ovaries (Choi et al., 2007; Joshi et al., 2007). In addition to these germ cell‐specific transcription factors, TAF4B, a subunit of the general transcription factor TFIID has been linked to POI in human, and the *Taf4b* knockout mice exhibited delayed CBD and excessive germ cell loss after birth (Grive et al., 2014). As for the importance and complexity of transcriptional regulation during CBD and follicle assembly, it still remains unclear how the transcriptional network orchestrates the whole process in such a critical window of development.

At the time of primordial follicle formation, the overall histoarchitecture of the ovary changes dramatically. The asynchronism of follicle assembly determines the heterogeneity of germ cells, and all three kinds of germ cells (germ cells in cysts, germ cells undergoing CBD, and germ cells in follicles) coexist in the newborn ovary (P0.5). Theoretically, changes in the transcriptomes of these germ cells can accurately represent the developmental transitions during follicle formation. Single‐cell transcriptome sequencing has recently become popular because it enables us to study the gene expression profiles in single cells. Different from previous study with ovarian tissues for genome‐wide expression profiles in rats (Kezele et al., 2005), in this study, we sequenced the transcriptome of 146 single germ cells collected from P0.5 ovaries, and our data clearly showed the temporal dynamics of germ cells on gene expressions. Further study identified the regulatory networks of transcription factors (TFs) and revealed how TFs and target genes coordinate the state transitions during follicle assembly.

## 2 RESULTS

### 2.1 RNA profiling of single germ cells from newborn (P0.5) ovaries

**FIGURE 1:**
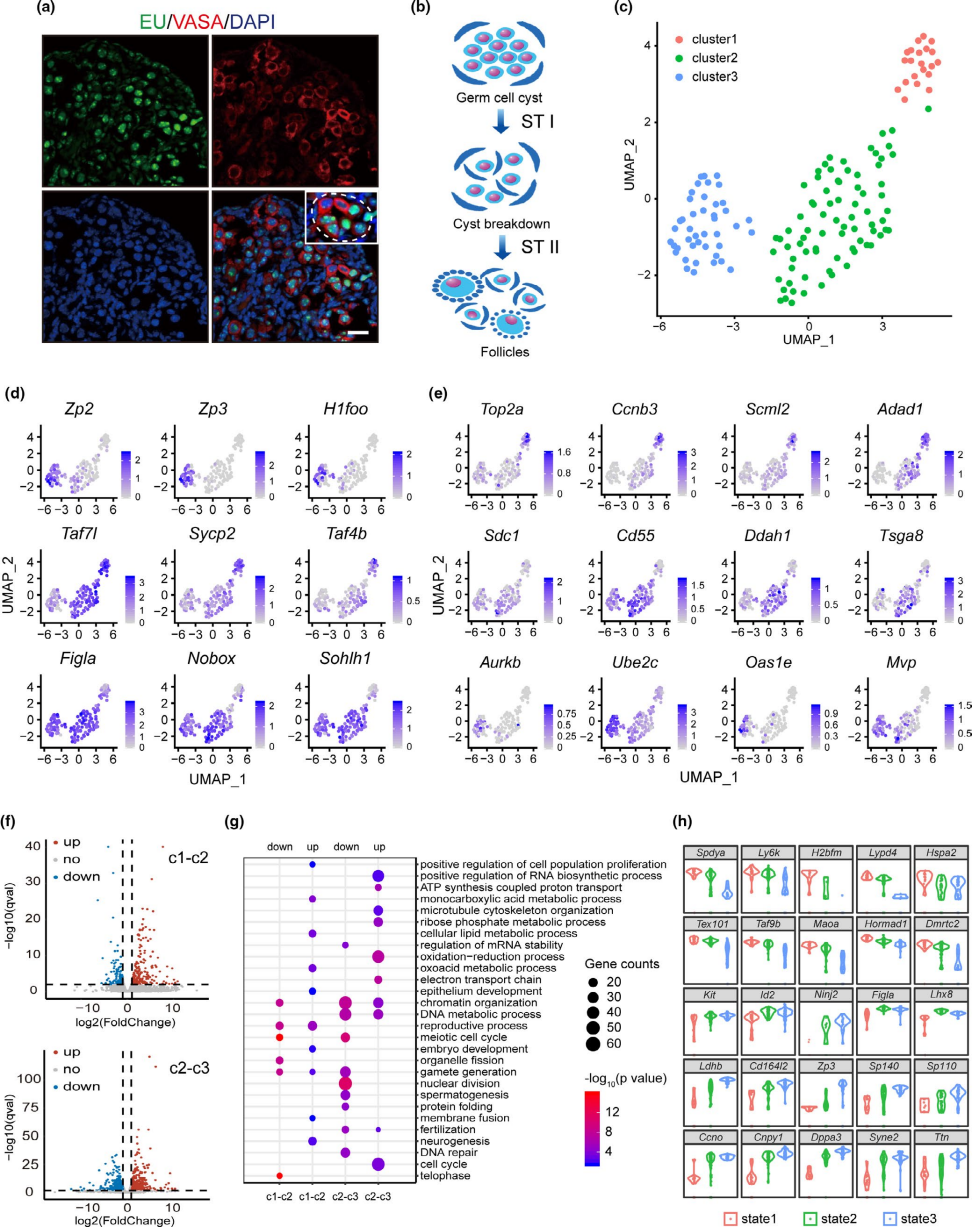
Single‐cell profiling reveals the heterogeneity in germ cells of newborn ovary. (a) EU labeling showed differentially transcriptional activities in germ cells of a newborn ovary (P0.5). P0.5 newborn mice were i.p. injected with EU (0.2 mg/each pup), and ovaries were collected 2 h later for immunostaining with antibodies against EU and VASA. Oocytes were labeled with VASA staining. Green, EU; Red, VASA; Blue, DNA. Inset, white dashed line circled a representative germ cell cyst. Bar = 20 μm. (b) Stage transitions from germ cell cysts to follicles. STI, from germ cell cyst (state 1) to cyst breakdown stage (state 2); STII, from cyst breakdown to follicle stage (state 3). (c) Cell clustering by UAMP based on gene expression. Cells from different clusters are shown in different colors. (d) Distributions of feature genes in clusters. The feature genes refer to genes selected to perform PCA analysis during cell clustering. The gene expression levels are indicated by the colors of the bar. (e) Identified DEGs with specific distributions of expression in three clusters. (f) Volcano Plots of differential expressed genes (DEGs) between clusters. (g) Representative GO terms of downregulated and upregulated enrichment between clusters. (h) Violin plots of enriched DEGs expression in three states. State 1 represents germ cells in cyst; state 2, germ cells undergoing cyst breakdown; and state 3, oocytes in follicles

It has been previously reported that asynchronism occurs in follicle assembly. The heterogeneity of germ cells in newborn (P0.5) ovary can be reflected by EU labeling, which indicates the different transcriptional statuses among germ cells (Figure 1a). At such a particular developmental stage, all three kinds of germ cells (germ cells in cysts, germ cells undergoing CBD, and germ cells in follicles) coexist in the newborn ovary, which represents two‐stage transitions as STI, state transition I, from cyst to CBD states and STII, state transition II, from CBD to follicle state (Figure 1b). To delineate the continuous nature of oocyte differentiation during follicle assembly, we collected single germ cells from P0.5 ovaries of C57BL6 mice by enzyme digestion and mouth pipetting. Germ cells are easily distinguished from somatic cells by their diameters (13~20 μm) under the microscope. We then analyzed the transcriptome of single germ cells by single‐cell RNA‐seq using Smart‐seq2 technology. The properties of oocytes were verified in advance by real‐time PCR after the preamplification of single‐cell mRNAs, and a total of 146 single germ cells were sent for sequencing. RNA‐seq libraries from single cells were sequenced to an average depth of 25 million reads, and 12,103 genes were identified, with confirmed expression in at least 10 single cells (TPM ≥ 0.1) (Table S1). After quality control, 142 cells were qualified for further analysis (Figure S1A).

### 2.2 Germ cells are molecularly heterogeneous

**FIGURE 2:**
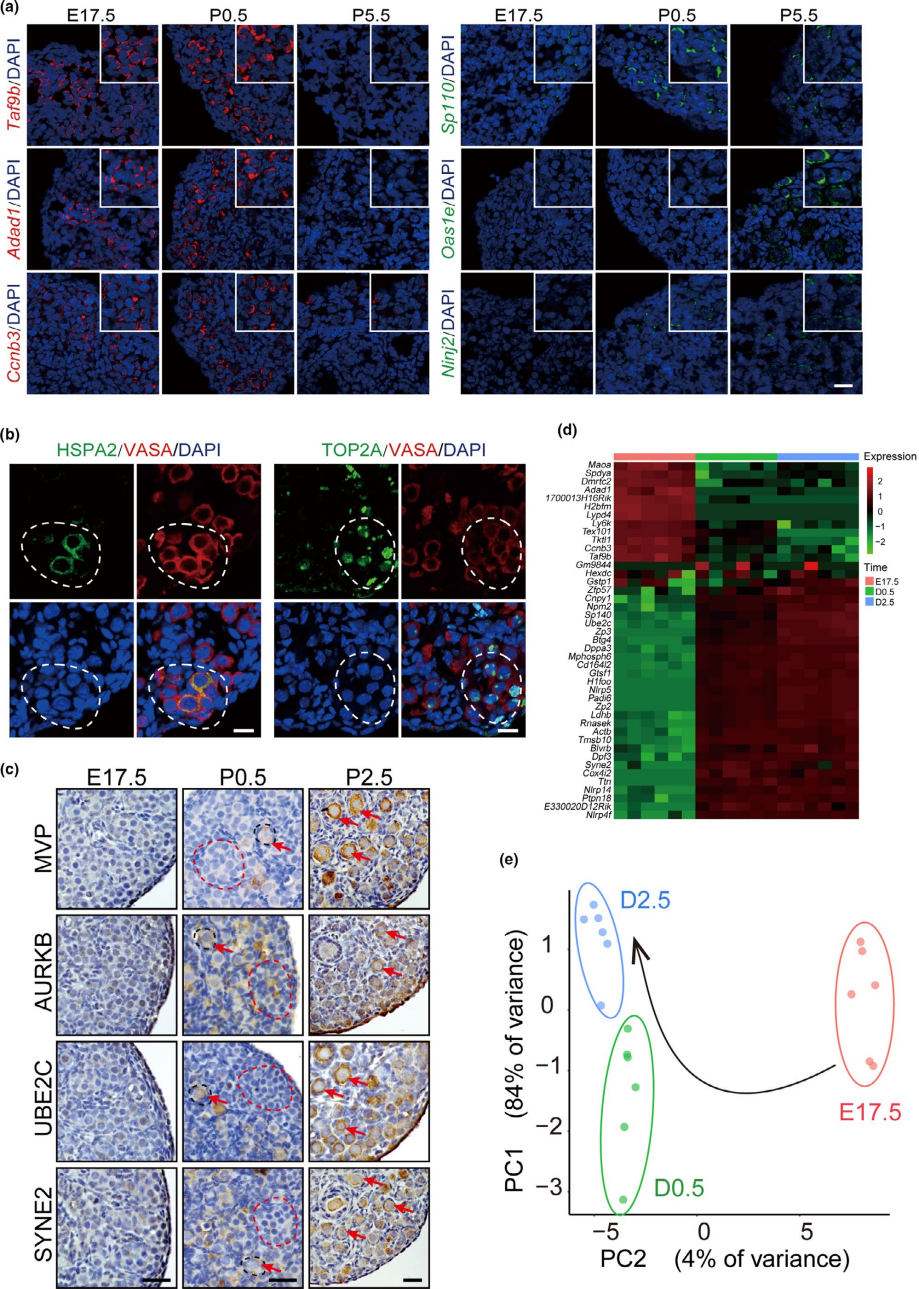
Representative gene expressions in newborn ovaries. (a) In situ hybridization of representative DEGs. Left panel, DEGs in STI from cyst to CBD stage. Right panel, DEGs in STII from CBD to follicle stage. Red and Green, specific probes of target genes; Blue, nuclear. Bar = 50 μm. Insets, magnified images to show the staining signals in germ cells/oocytes. (b) Differential expression of representative downregulated genes TOP2A and HSPA2 in germ cells of P0.5 ovary. Germ cells were labeled with VASA staining. Red, VASA; Green, specific proteins; Blue, nuclear. White dashed lines circled germ cell cysts. Bars = 20 μm (c) Dynamic expression of representative upregulated genes in E17.5, P0.5, and P2.5 ovaries. Red dashed lines circled germ cell cysts; Black dashed lines circled primordial follicles. Oocytes in follicles were labeled with red arrows. All bars = 50 μm. (d‐e) High‐throughput qPCR profiling of state DEGs (43 genes) in germ cells/oocytes collected from E17.5, P0.5, and P2.5 ovaries. Hierarchical clustering ( d) and PCA plotting ( e) showed three distinguishable cell groups correlated to their developmental stages. Red, E17.5; Green, P0.5; Blue, P2.5

To reveal the heterogeneity (subpopulation) of germ cell development during follicle assembly, we first clustered the cells into three subtypes (clusters 1, 2, and 3) using Uniform Manifold Approximation and Projection (UMAP) method according to the expression of well‐known genes that are important in oocyte development (Figure 1C and Table S1). *Sycp2*, *Stag3*, and *Taf7l* are genes with high expression in fetal meiotic germ cells. Accompanied with the arrest of meiosis at the diplotene stage of prophase I after follicle formed, their expressions will decrease significantly in oocytes (Fukuda et al., 2014). *Sohlh1*, *Nobox*, *Figlα*, and *Lhx8* are the genes important for follicle formation as knockout mice of either one has the defects on oocyte survival and follicle assembly (Choi et al., 2007; Grive et al., 2016; Joshi et al., 2007). *Kit*, *Gdf9*, *Zp1*, *Zp2*, *Zp3*, *H1foo*, *Ooep*, *Nlrp5*, *Nlrp14*, *Pou5f1*, *Ybx2*, and *Zar1* are well‐known maternal genes for oocyte and early embryonic development (Park et al., 2015). As shown in Figure 1d and Figure S1b, these genes showed differential distributions in clusters. Additionally, we also identified more differential expressed genes (DEGs) in comparison between adjacent clusters (Figure 1e and Figure S1c). In total, we identified 141 upregulated and 71 downregulated genes between cluster 1 and cluster 2, and 433 upregulated and 492 downregulated genes (q < 0.001) were recognized between cluster 2 and cluster 3 (Table S2 and Figure 1f), respectively. GO analysis revealed male meiosis and synapsis terms in downregulated genes from cluster 1 to cluster 3 (Figure 1g). Besides gametogenesis or embryo development processes, neurogenesis, membrane fusion terms were specifically enriched in upregulated genes between cluster 1 and cluster 2, and electron transport chain, ATP synthesis, oxidation‐reduction process etc. were specifically enriched in upregulated genes from cluster 2 to cluster 3. Such processes are correlated with dramatic cell motility and cell‐cell communications during follicle assembly, and the metabolic changes after follicle formed. In mice, oocyte meiosis starts at approximately E13.5, progresses through the leptotene, zygotene, and pachytene stages, and finally arrests at the diplotene stage of prophase I when the follicle is formed. The oocytes then remain in diplotene until the first meiotic division just prior to ovulation. Thus, from cluster 1 to cluster 3, the downregulation of meiosis‐related genes and the significant upregulation of genes for oocyte and embryonic development represent the stage‐to‐stage transitions of germ cells from cyst to follicle stage. The three cell clusters correspond to three germ cell types in the newborn ovary which we referred as state 1, germ cells in cyst (with the highest expression of meiosis‐related genes); state 2, germ cells undergoing CBD (transitional state); and state 3, germ cells formed follicles (with the highest expression of genes related with oocyte or embryonic development). Violin plots revealed the distributions of DEGs between states, and some representative genes were manifested by in situ hybridization, real‐time RT‐PCR, and Western blot on ovaries collected from E17.5, P0.5, P2.5, or P5.5 (Figure 1h and Figure S1d; Figure 2a; Figure S2a and 2b). Immunofluorescence of TOP2A and HSPA2, the genes with high expressions in state 1 cells, showed differential expressions in germ cells of the same cyst in P0.5 ovaries (Figure 2b). The dynamic expression of MVP, AURKB, SYNE2, and UBE2C was also observed in E17.5, P0.5, and P2.5 ovaries with the dramatic increase in oocytes formed follicles (Figure 2c). Cell suspension was then prepared from E17.5, P0.5, or P2.5 ovaries, and oocytes were picked by mouth pipette for high‐throughput on‐chip quantitative real‐time PCR with selected DEGs identified between state transitions (Figure 2d; Table S3). After PCA analysis, the cells can be clearly clustered into three clusters according to the expression levels of these genes (Figure 2e).

### 2.3 Reconstruction of the temporal dynamics of germ cells during follicle assembly

**FIGURE 3:**
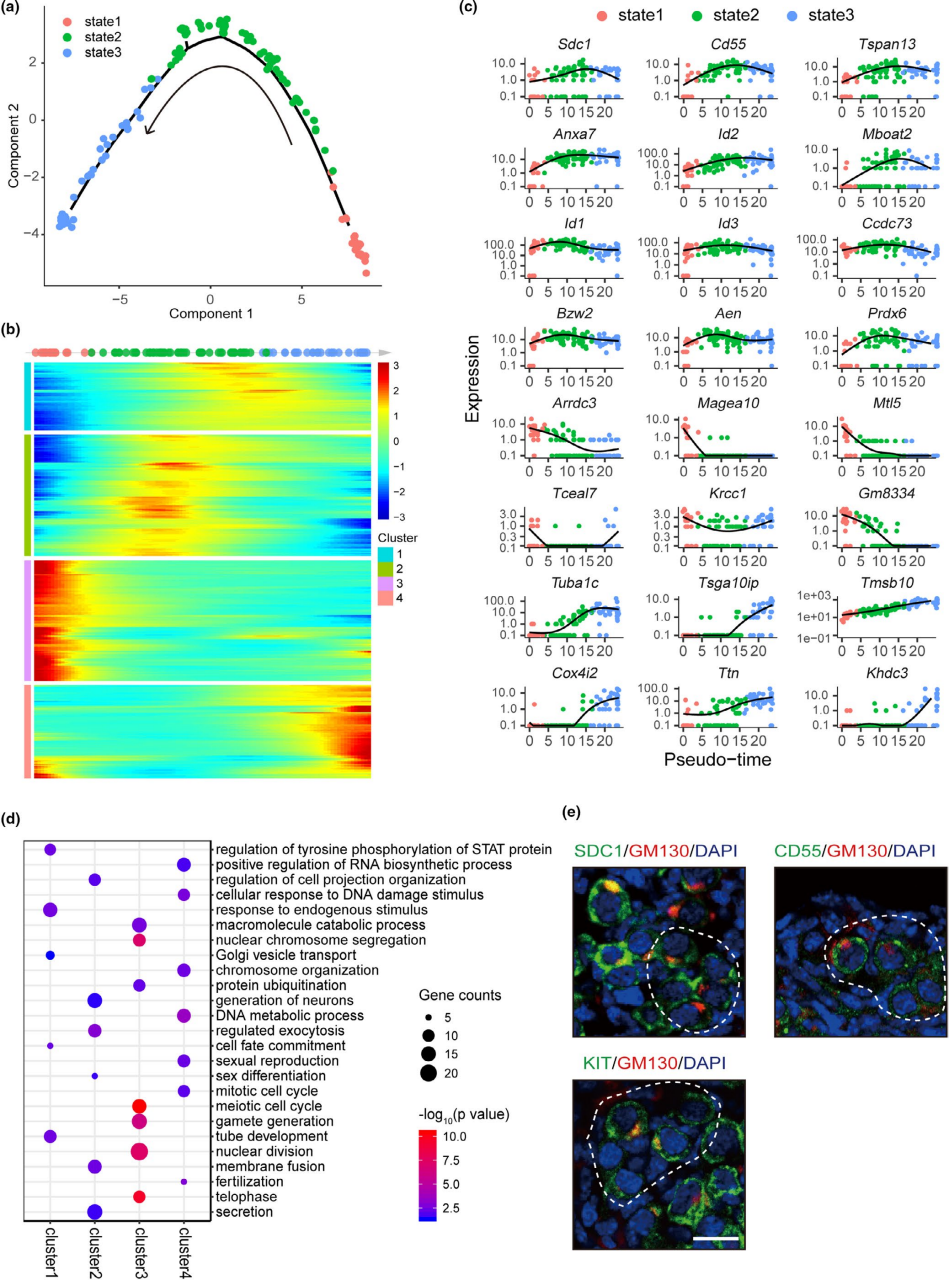
Reconstruction of the temporal dynamics in germ cell development. (a) Monocle analytic plot of germ cells in 2D‐PCA space, using a minimum spanning tree. The beginning and end of the timeline were determined by the known marker genes. (b) Heatmap representing the smoothed expression of pseudo‐time‐dependent genes alone pseudo‐timeline. Pseudo‐time‐dependent genes were clustered into 4 clusters according to different expression patterns. (c) Expression trend of selected marker genes along the pseudo‐timeline. (d) List of the most relevant GO terms in each cluster of pseudo‐time‐dependent genes together with the enrichment significance scores. (e) Co‐localization of identified membrane proteins SDC1, CD55, with GM130 in newborn P0.5 ovaries. KIT and GM130 staining were used as positive control to label oocytes destined to form follicles. White and red dashed lines circled germ cell cysts; black dashed lines circled primordial follicles. All bars = 20 μm

One of the advantages of single‐cell transcriptional profiling is to order cells along a hypothetical timeline of development by pseudo‐time analysis. To delineate the temporal dynamics of germ cells during follicle formation, Monocle toolkit was used to reorder single cells into a pseudo‐temporal timeline, and the result clearly demonstrated the uniform development of germ cells from cyst (state 1) to follicle stage (state 3) (Figure 3a). The heatmap showed the trends of pseudo‐time‐dependent genes along the pseudo‐timeline which were then classified into four clusters with different expression dynamics (Figure 3b and Table S4). The trends of single genes in each cluster were plotted as shown in Figure 3c and Figure S3a. Genes in cluster 1 and cluster 2 showed tide–wave trend along the pseudo‐timeline (Figure 3b). GO analysis enriched generation of neurons, regulated exocytosis, cell fate commitment, or Golgi vesicle transport terms, which are in accordance with the dramatic organelle movement between oocytes and oocyte‐somatic interactions during follicle assembly (Figure 3d). Notably, *Figlα*, *Sohlh1*, *Nobox*, and *Lhx8*, all the mentioned transcription factor genes important for follicle formation are enriched in these two clusters. *Foxo3*, the forkhead transcription factor gene that is important for the maintenance of primordial follicles, is also enriched in cluster 2 (Reddy et al., 2010). Besides that, we also identified several genes encoding membrane proteins such as *Sdc1*, *Cd55*, *Tspan13*, and *Anxa7* (Figure 3c). We then checked the coexpression patterns of CD55 and SDC1 with GM130, a Balbiani body marker labeling oocytes that are destined to form follicles (Lei & Spradling, 2016). Meanwhile, KIT staining was used as a control because of its critical role in regulating the cross‐talk between oocytes and somatic cells during perinatal CBD. The results clearly showed their co‐localization with GM130‐positive germ cells (Figure 3e). These identified cell surface molecules may be used as markers to label and isolate oocytes that are destined to form follicles. Cluster 3 genes showed constantly downregulated trend along the pseudo‐timeline, and GO terms are related with meiotic cell cycle regulation (Figure 3c,d; Figure S3a). Cluster 4‐enriched genes, playing important roles in DNA metabolic process, fertilization, or miotic cell cycle, include many oocyte‐specific genes or maternal factors that are important for ovulation or early embryonic development such as *Zp1*, *Zp3*, *Btg4*, *Npm2*, *Padi6*, *Nlrp14*, and *Ooep* (Figure 3c,d; Figure S3a). Thus, from the dynamic gene expression along the pseudo‐timeline, we recaptured the sequential and stepwise trajectory of germ cell development and identified a series of pseudo‐time‐dependent genes that may function during primordial follicle formation and following follicular development.

### 2.4 Reconstruction of transcriptional modules related to cell states during follicle assembly

**FIGURE 4:**
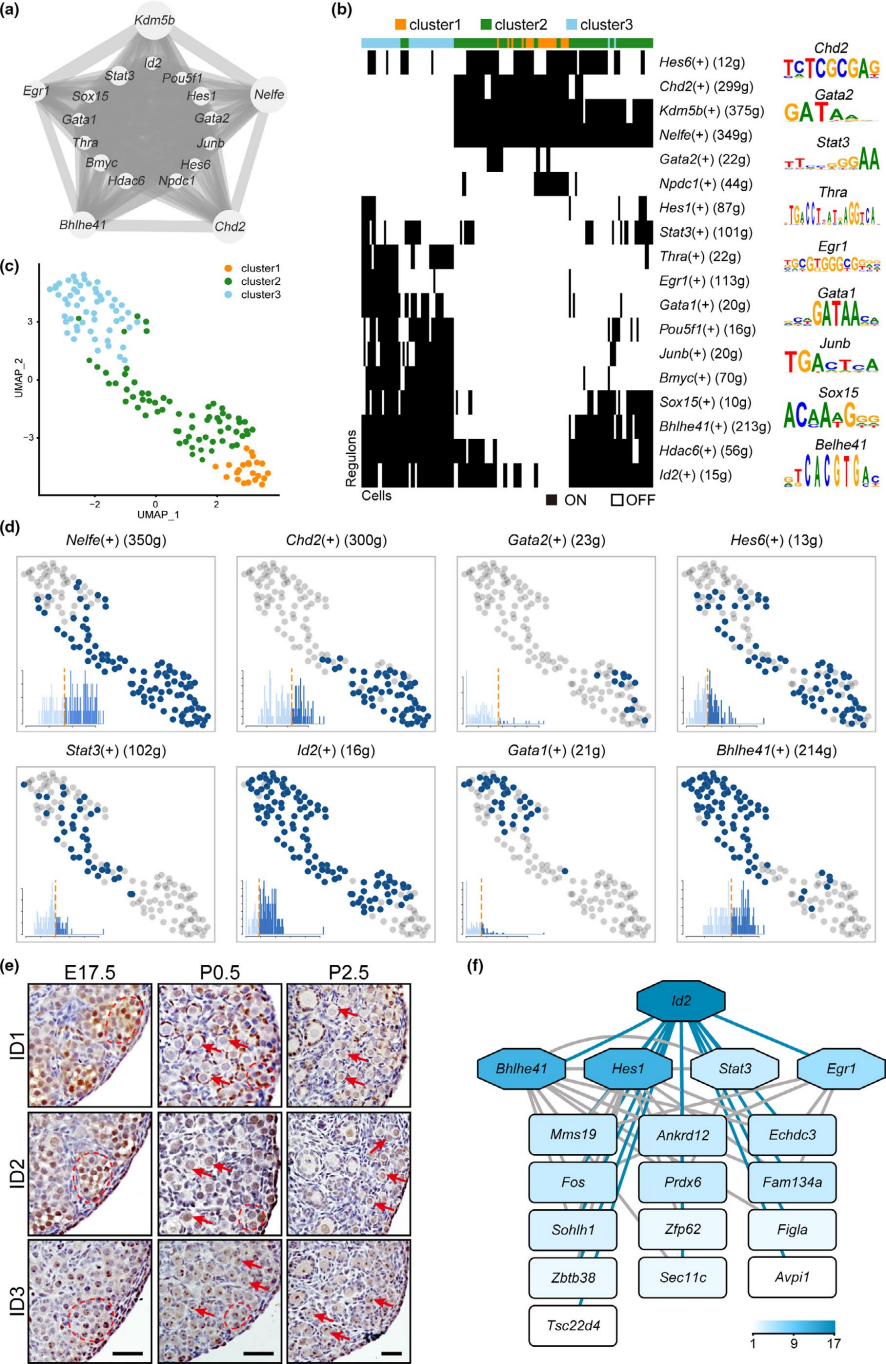
Analysis of transcriptional regulatory network. (a) Visualization of key regulons in regulatory networks. The node size indicates the number of target genes associated with corresponding transcription factor. The edge size indicates the weight of the connection. (b) Binary active heatmap (left) shows the activity of regulons in each cell. The color in black is active and in white is inactive. The number of target genes is given in parentheses next to each regulon. The enriched motif logos are shown in right. (c) Cell clustering based on regulon activity scores. (d) Binary active distribution of regulons. The states of regulons in each cell are indicated in dark blue (active) and gray (inactive). The histograms show the distribution of regulon active scores. (e) Dynamic expressions of ID1, ID2, and ID3 in ovaries at different developmental stages (E17.5, P0.5, and P2.5). Germ cell cysts are circled with red dashed lines and oocytes in follicles are labeled with red arrows. Bars = 50 µm. (f) Network of transcription factor and target genes in *Id2* regulons. The depth of the color represents the number of connections

To identify the master regulators of follicle formation, we constructed transcriptional regulatory networks with transcriptional regulators and their target genes by applying SCENIC (single‐cell regulatory network inference and clustering) analysis. The DEGs between stage‐transitions were input for SCENIC to set up the regulatory network. We identified 18 significant regulons containing 868 genes (Figure 4a and Table S5). The size of each regulon varies from 10 to 375 genes, with a median size of 44 genes. The regulon activity matrix revealed their differential expression between cell states (Figure 4b). Strikingly, cells are well separated into three states using the RAS‐based distance, and the cells showed highly overlapped distributions with previous clusters (Figure 4c and Figure S4a). The differential expressions of the regulons in states were then plotted (Figure 4d and Figure S4b). Among the enriched regulons, *Nelfe* and *Chd2* genes showed similar expression patterns in state 1 and state 2 cells (Figure 4d), and their enriched target genes were highly overlapped with each other (Figure S4c and Table S5), with enriched GO terms specific on nuclear division and chromosomal organization of meiotic cell cycle (Figure S4d). *Nelfe*, negative elongation factor E, has been previously identified as a RNA‐binging protein that participated in regulating the stability of the mRNA of MYC‐associated genes (Dang et al., 2017). Based on the highly overlapped target genes between *Nelfe* and *Chd2*, there is a possibility that *Nelfe* also modulates *Chd2*‐related genes in oocyte during follicle formation. *Bhlhe41*, which is highly expressed in follicle oocytes (state 3 cells), together with the other two identified core regulons *Hes1* and *Hes6*, are components of Notch signaling pathway. The importance of Notch signaling pathway in follicle formation has been well delineated in previous studies (Vanorny et al., 2014). GO analysis of *Bhlhe41* target genes further revealed enriched biological processes about neurogenesis and cellular component organization (Figure S4e). The result suggests the regulation of *Bhlhe41* on cell–cell communications during follicle assembly is possibly through the Notch signaling pathway. The activity of another transcription factor, *Id2*, was enriched in state 2 and state 3 cells, showed nuclear location in germ cells/oocytes of perinatal ovaries (Figure 4d,e). The regulatory network of *Id2* with its target genes was shown as Figure 4f in which we surprisingly found that most of these target genes are transcription factors. It not only included the identified regulons *Bhlhe41*, *Stat3*, *Hes1*, and *Egr1*, but also oocyte‐specific transcription factors, *Sohlh1* and *Figla*. The results suggest *Id2* may function in oocyte development through the regulations on target TFs. To be noted, the other two ID family members ID1 and ID3 were also enriched as DEGs between stage transitions. As validated by immunostaining on ovaries at different developmental stages (E17.5, P0.5, and P2.5) (Figure 4e), the three IDs underwent differential and dynamic expression changes during follicle formation. Recently, a study on single‐cell sequencing of human fetal germ cells (FGCs) also identified these three IDs, and associated them with the transition stage from mitosis to mitotic arrest in FGCs (Zhang et al., 2018). The dynamic expressions of three IDs imply their differential regulation on follicle formation. Furthermore, our result highlights the role of ID2 during the process.

### 2.5 Regulation of ID1 and ID2 in the assembly of primordial follicles

**FIGURE 5:**
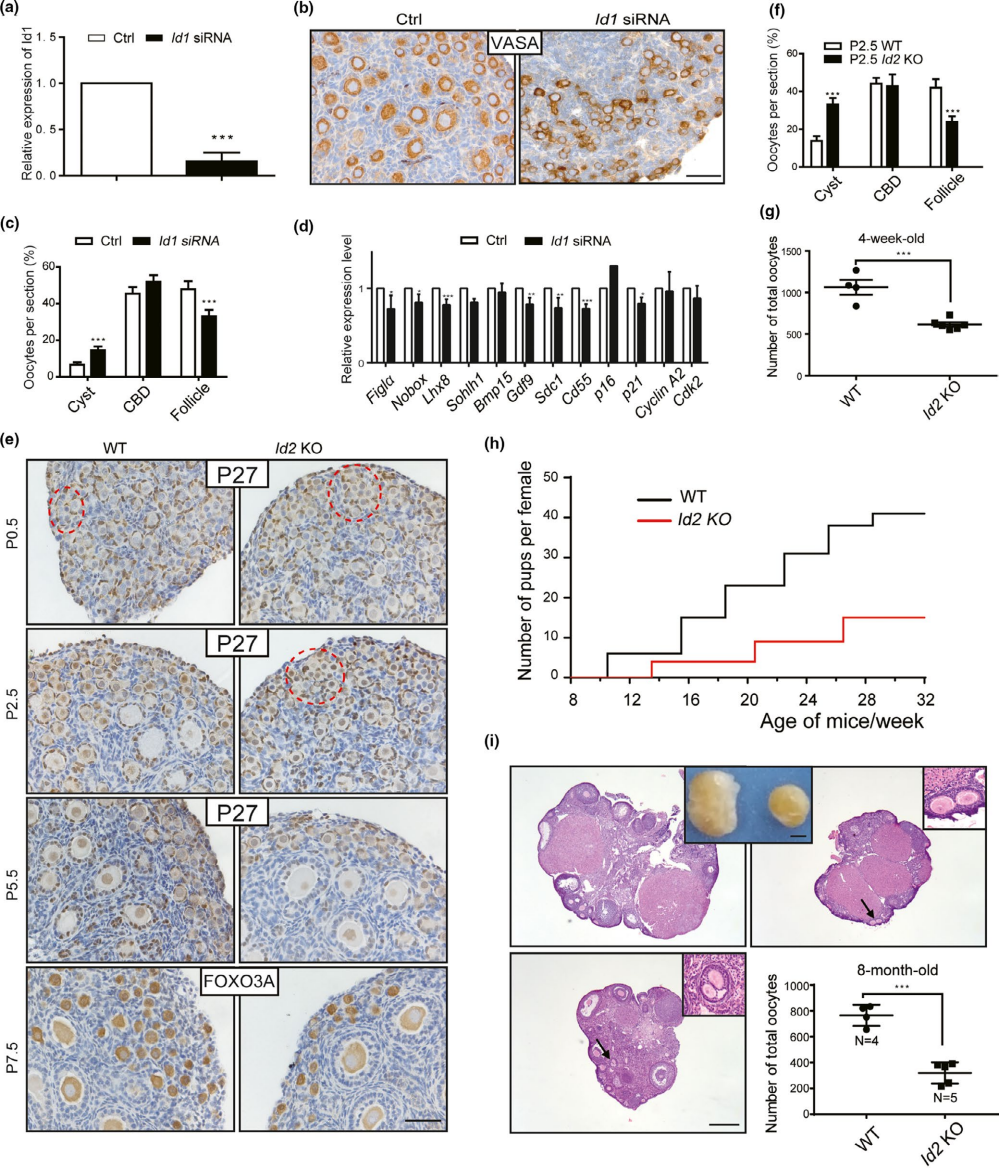
Effects of ID1 or ID2 on primordial follicle formation. (a) Efficiency analysis by RT‐qPCR after knockdown of *Id1*. (b and c) Inhibition of follicular formation by *Id1* siRNA treatment. (b) Oocytes were labeled with VASA staining; (c) Distribution of oocytes in germ cell cyst (Cyst), cyst breakdown (CBD), and follicle. (d) Effects of *Id1* knockdown on ovarian gene expressions. The levels of all tested mRNAs in the control group were set to 1. (e and f) Ovarian development in wildtype (WT) and *Id2* KO mice. (e) P27 and FOXO3A staining were used to label oocytes and follicles. (f) Distribution of oocytes in P2.5 WT and *Id2* KO ovaries. (g) Decreased total oocyte number in 4‐week‐old *Id2* KO ovaries. All bars = 20 µm. (h) Comparison of the cumulative numbers of pups per female in WT (black) and *Id2* KO mice (red). (i) Ovarian morphology, histology, and follicle counting of 8‐month‐old WT and *Id2* KO mice. Ovarian histology shown by H&E staining was collected from different *Id2* KO mice. Bars = 400 µm. All data were presented as mean ±SD of at least three repeats. *, *p* < 0.05; **, *p* < 0.01; ***, *p *< 0.001

Inhibitors of DNA‐binding (ID) family members, including ID1, ID2, ID3, and ID4, belong to the helix‐loop‐helix (HLH) family of transcription factors and are key regulatory proteins in a wide range of developmental and cellular processes (Perk et al., 2005). Following detection of the dynamic and differential expression of three IDs (ID1, ID2, and ID3) in newborn ovaries, we next sought to determine their functions in the formation of primordial follicles. Similar to a previous study, our study showed that *Id3* KO female mice displayed normal follicular development, and the mice were fertile during a 6‐month breeding test (data not shown) (Pan et al., 1999). However, a delay of cyst breakdown and follicle formation was observed when P0.5 ovaries were incubated with *Id1* siRNA for 4 days (Figure 5a‐c). RT‐PCR results revealed a significant decrease in the oocyte development genes *Figla*, *Nobox*, *Lhx8*, and *Gdf9*. Other genes that showed dramatic changes are *Cd55* and *Sdc1*, the membrane‐related genes identified in the study, and *P16* and *P21*, the cyclin‐dependent kinase inhibitors regulated by IDs (Figure 5d) (Ling et al., 2014).

It has been reported that *Id2* KO mice lacked lymph nodes and Peyer's patches, and adult female KO mice could deliver pups, but all pups died in 2 days because of lactation defects in mammary development (Miyoshi et al., 2002; Yokota et al., 1999). To evaluate the effect of *Id2* on early follicular development, ovaries at different developmental stages were collected from *Id2* KO mice. We used P27 staining to evaluate follicle assembly in neonatal ovaries and FOXO3A staining to distinguish primordial follicles from growing follicles after follicle formation (Rajareddy et al., 2007; Reddy et al., 2010). Compared with normal ovarian morphology in P0.5 KO ovaries, a delay in follicle formation was observed in P2.5 KO ovaries (Figure 5e,f. Following observations revealed continuous follicle formation at P5.5, with only a few germ cells left in cysts of KO ovaries (Figure 5e, P5.5). In P7.5 KO ovaries, although follicular activation and development were not affected, fewer primordial follicles were observed in the ovarian cortex (Figure 5e, P7.5). Follicle counts of ovaries from 4W KO mice further demonstrated a dramatic decline on total follicle numbers (Figure 5g). However, follicle distributions were not affected between the two groups. Adult female WT or KO mice (8W) were then mated with WT fertile males for 6 months, and KO mice exhibited subfertility with longer between‐labor intervals and decreased frequency of litters (Figure 5h). After mating trials ended, ovaries were immediately collected and significant decreases in ovarian size were found in KO mice (Figure 5i). Histology analysis demonstrated the existence of follicles at different developmental stages in both WT and KO ovaries except that multi‐oocyte follicles occasionally appeared in KO ovaries (Figure 5i, insets). Follicle counting result again revealed a critical reduction in total follicle number in KO mice (Figure 5i). The result suggests the sharp reduction in follicle numbers causes the subfertility phenotype.

**FIGURE 6:**
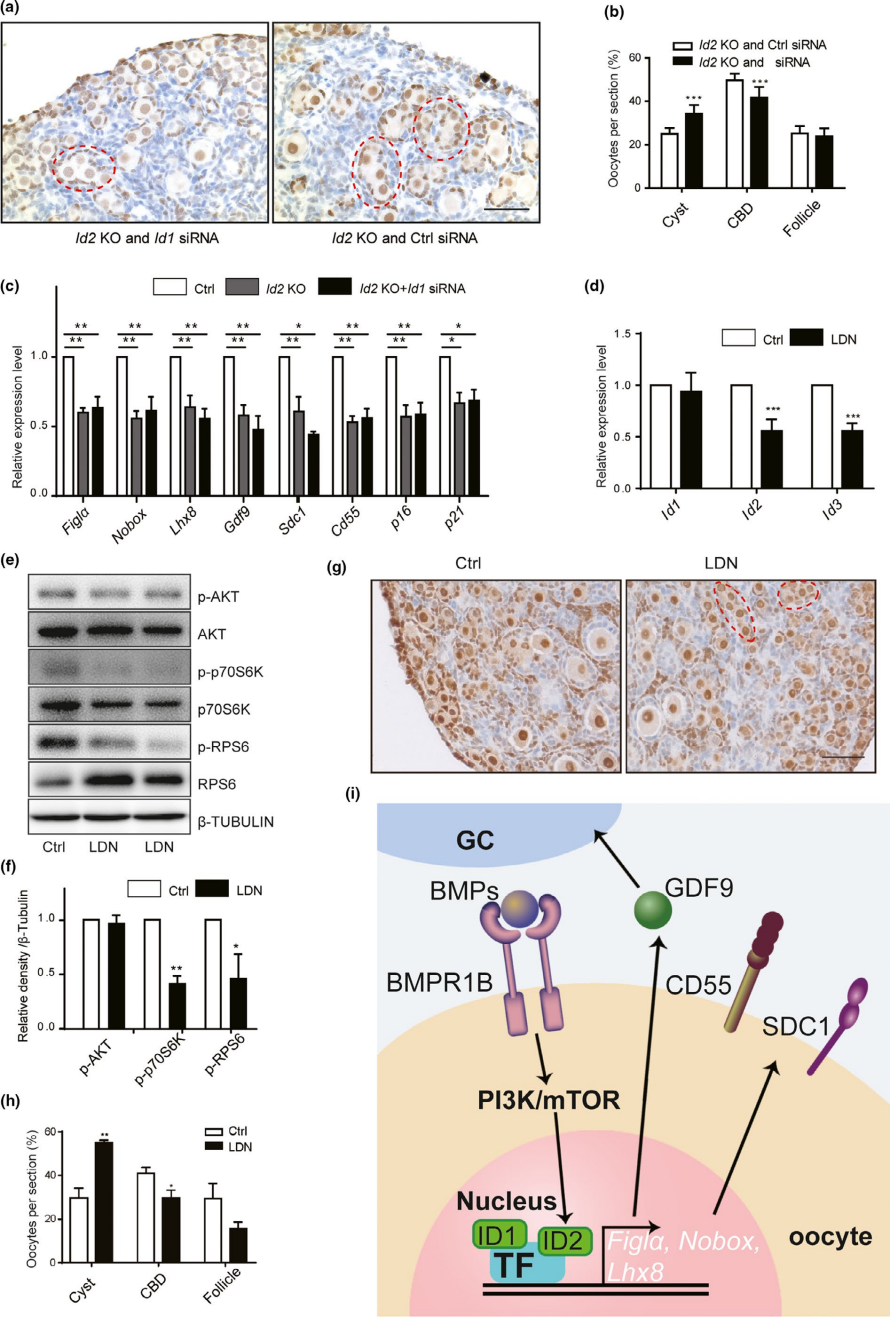
Differential regulation of *Id1* and *Id2* on follicle assembly. (a and b) Knockdown of *Id1* in *Id2* KO ovaries. (a) oocytes and follicles were labeled by P27 staining. (b) Distribution of oocytes in germ cell cyst (Cyst), cyst breakdown (CBD), and follicle. (c) Effects of *Id1* knockdown on expression of selected genes in *Id2* KO ovaries. (d) Blocking BMPR1B by its specific inhibitor LDN193189 reduced the expression of *Id2* and *Id3* but not *Id1*. (e and f) Western blotting (e) and densitometry quantification (f) showed the downregulation of PI3K/mTOR signaling pathway after LDN193189 treatment. (g and h) Inhibition of follicle assembly by LDN193189. G, P27 staining. (h) oocyte distributions. (i) The diagram of the roles of ID1 and ID2 in primordial follicle formation. BMP signaling functions through its membrane receptor BMPR1B and activates the PI3K/mTOR signaling pathway which results in the increased expression of *Id2* and its downstream‐regulated genes. ID1 also participates in the follicle assembly but in a BMP‐independent manner. Bars = 20 µm. All data were presented as mean ± SD of at least three repeats. The levels of all tested mRNAs in the control group were set to 1. *, *p* < 0.05; **, *p* < 0.01; ***, *p* < 0.001

To determine whether additive effects existed between ID1 and ID2 in follicle formation, we collected newborn (P0.5) ovaries from *Id2* KO mice and incubated them with or without *Id1* siRNA for 4 days. Compared with that in *Id2* KO mice, the addition of *Id1* siRNA further compromised the process of CBD, with more germ cells kept in cysts after the treatment (Figure 6a,b). However, when RT‐PCR was performed to check the expression of genes regulated by *Id1* siRNA, no synergistic effects were found (Figure 6c). Several *Id* genes have been shown to be activated by bone morphogenic proteins (BMPs) via SMADs in a variety of cell types (Perk et al., 2005). Because BMPR1B, the type I receptor for BMP4 and BMP7, was also identified as the DE gene in our single‐cell sequencing data, we subsequently treated newborn ovaries (P0.5) with its specific inhibitor LDN193189 for 4 days. The inhibition of LDN193189 on follicle assembly was manifested by ovarian morphology and follicle counting results (Figure 6g,h). However, RT‐PCR result only showed significant decreases in *Id2* and *Id3* mRNAs (Figure 6d). Meanwhile, the inhibition of the PI3K/mTOR signaling pathway was also observed in LDN193189 treated ovaries (Figure 6e,f). Due to the critical role of the PI3K/mTOR signaling pathway in follicle formation (Zhang et al., 2017), we concluded that BMPs regulated *Id2* through the activation of the PI3K/mTOR signaling pathway. Taken together, both ID1 and ID2 are involved in the process of follicle formation but play a role in different regulatory mechanisms (Figure 6i).

## 3 DISCUSSION

The recent development of single‐cell sequencing methods has led to multiplexed sampling of cellular states within a tissue. In mammals, CBD and follicle formation is an important process to regulate oocyte numbers. During the process, not all of the oocytes within the cysts survive to form follicles, the fate of oocytes that are incorporated into primordial follicles is controlled by the oocyte (Sarraj & Drummond, 2012). Here, in contrast to previous studies of whole ovaries from different developmental stages, the uniform development of oocytes can be well reflected in the newborn ovary by single‐cell gene expression profiling. The significant decrease in meiosis‐ or spermatogenesis‐related genes and the dramatic increase in oocyte‐specific genes that are important for oocyte and embryonic development mark the transition from germ cells to functional oocytes. Our data also highlighted the complexity of transcriptional control and dissected variability in RNA splicing events during the process, and furthermore, depicted the regulatory networks in state transitions along the developmental timeline (Figure S5).

To establish, the structure of primordial follicles requires the mutual communication between oocytes and the surrounding somatic cells. The assembly of primordial follicles in mouse starts from medullar region and gradually extends to the cortical region with two kinds of primordial follicle formed: the first wave primordial follicles in medulla and the adult primordial follicles in cortex. The two kinds of primordial follicles are distinguished by the sequential expression of *Foxl2* (Forkhead box L2) in pregranulosa cells and have distinct developmental dynamics and play different roles in mammalian reproductive lifespan (Zheng et al., 2014). Despite the developmental difference in the two kinds of primordial follicles, in our study, the Monocle analysis only revealed uniform development of germ cells. The result suggests the concomitant control of somatic cells on follicle assembly and its determination on oocyte developmental fate once follicle formed. Notch signals have been well demonstrated as one of the most important signaling pathways connecting the two cell types during follicle formation. Conditional deletion of *Jag1* in germ cells and *Notch2* in granulosa cells both resulted in multi‐oocyte follicles due to incomplete germ cell cyst breakdown (Vanorny et al., 2014). In the study, *Jag1* was specifically enriched at the transition from state 2 to state 3. Moreover, SCINEC analysis identified components of Notch signaling pathway, *Bhlhe41*, *Hes1*, and *Hes6* as transcriptional regulons during the process. The result further revealed the transcriptional regulation of Notch signaling pathway in follicle assembly. Besides Notch signals, another important signaling pathway, KIT signaling pathway, has been demonstrated to promote germ cell cyst breakdown and determine oocyte numbers (Jones & Pepling, 2013). Different from *Jag1*, *Kit* was specifically enriched at the stage transition from state 1 to state 2. Thus, in accordance with previous studies, our study further revealed the sequential events that occur during follicle formation: KIT signals initiate cyst breakdown and determine the oocyte numbers, and Notch signals function subsequently to direct the assembly of primordial follicles through contacts between the two cell types.

It is well accepted that oocytes are functionally immotile during primordial follicle formation, whereas pregranulosa cells are motile, allowing invasion under the direction of oocytes (Zhang et al., 2017). In addition to oocyte differentiation, the final size of the ovarian reserve is also determined by the proper differentiation and proliferation of ovarian supporting cells such as the recruited number of pregranulosa cells and speed of recruitment. Similar to oocytes of diverse species, mouse oocytes differentiate by receiving organelles from neighboring sister cyst germ cells, and this process is microtubule dependent (Lei & Spradling, 2016). Those germ cells that receive organelles from neighboring cyst cells, increase in size and build a Balbiani body to become oocytes finally, whereas nurse‐like germ cells die through apoptosis. The finding suggests the initiative oocyte selection by active organelle transfer between sister‐germ cells. Here, many genes related to the microtubule/actin cytoskeleton were upregulated along the pseudo‐timeline. Meanwhile, regulatory networks involved in secretion, exocytosis, generation of neurons etc. were also enriched with tide expression pattern along the pseudo‐timeline. Such gene expression patterns coincide with active cell migration and dramatic structure establishment during follicle assembly. Through analyzing the gene expression dynamics in single germ cells, our results could not only accurately reflect the interactions between germ cells but also reveal the oocyte‐somatic or oocyte‐ECM communications. Future study with two kinds of pregranulosa cells will be better in delineating the dialogues between germ and somatic cells.

In most female mammals, it has been well accepted that significant germ cell loss occurs prior to and during follicular assembly. Although the exact underlying mechanism remains unknown, it is well accepted that this takes place through apoptosis, one type of programmed cell death (PCD) mechanisms. The balance between BCL‐2 proteins, mainly BCL‐X and MCL‐1, and BAX is believed to determine the death or survival of germ cells (Sun et al., 2017). Additionally, the activation of a p63/p53‐ and PCNA‐dependent checkpoint also plays a major role in eliminating defective oocytes during CBD and follicle formation (Klinger et al., 2015). In this study, our single‐cell sequencing data revealed the uniform development of germ cells during follicle assembly. Surprisingly, we did not find a large number of “dying” oocytes as anticipated. We reason this to the sequencing technique for surviving cells and all apoptotic/dead cells will be removed at the first steps of quality control during sequencing. Another reason is related with the asynchronous occurrence of apoptosis in oocytes during CBD and follicle formation. This could be manifested by the scattered and few TUNEL or BAX signals observed in perinatal ovaries (Greenfeld et al., 2007). However, our data enriched the development‐delayed germ cells (state 1) with significant decreased expression of oocyte survival genes, such as *Figla*, *Nobox*, *Sohlh1*, or *Lhx8*. According to the proposed theory, these cells that fail to form follicles will be deleted finally. Our study also identified *Tp63* as the DE gene, and it was then found as the target gene of enriched transcription regulons *Gata1* or *Chd2*. As the only member of p53 family expressed in oocyte nucleus, p63, especially its isoform TAp63, starts to express in the diplotene stage beginning around E18.5 and its expression level keeps high in growing follicles during ovarian development. *p63* deletion has no obvious effect on primordial formation; however, its null mutation prevents radiation‐induced oocyte apoptosis (Livera et al., 2008). Although we still do not know the regulatory mechanism on TAp63 expression during the process, our result again revealed the surveillance of the p63 system on survival oocyte numbers through its regulation on apoptosis.

As the helix‐loop‐helix (HLH) family members, ID proteins harbor a HLH motif which mediate dimerization with other basic HLH proteins, primarily E protein transcriptional factors. However, because they do not possess the basic amino acids adjacent to the HLH motif necessary for DNA binding, the binding of ID proteins with E proteins normally inhibits their functions by preventing them from binding DNA or forming active homo‐ or heterodimers (Ling et al., 2014). Besides their redundant functions in sequestering the E proteins, the ID proteins have also been shown to interact with other proteins without the HLH motifs and function differentially in a variety of cell types (Perk et al., 2005). Loss of *Id1* and *Id3* during embryogenesis leads to premature neural differentiation and poor vascularization, whereas loss of *Id2* leads to an alteration of cell fate in mammary (Lyden et al., 1999; Miyoshi et al., 2002). In the study, the three ID family members, *Id1*, *Id2*, and *Id3* were all identified as DEGs in the newborn ovaries, but immunohistochemistry showed their distinct expression patterns in ovaries at different developmental stages. Following functional studies showed the regulations of ID1and ID2, but not ID3 on CBD and follicle assembly. Although synergistic effects on follicle formation were found in *Id2* KO ovaries treated with *Id1* siRNA, after newborn ovaries being treated with BMP inhibitor LDN193189, it only showed the reduction in *Id2* expression. The result suggests the differential regulation of *Id1* and *Id2* during primordial follicle formation. BMP members BMP4 and BMP7 have been reported to promote primordial to primary follicle transition and a BMP4 antibody dramatically reduces the number of primordial follicles in the rat (Lee et al., 2004; Nilsson & Skinner, 2003). Recently, one study showed stromal cell‐specific knockout *Senp1*, a small ubiquitin‐related modifier (SUMO)‐specific isopeptidase, attenuated follicle formation by markedly downregulated expression of BMP4 (Tan et al., 2017). Here, our study not only identified *Bmpr1b* as the DE gene and *Id2* as the key regulon in oocytes but also demonstrated the regulation of BMPs on ID2 through the PI3K/mTOR signaling pathway. From the phenotype of *Id2* KO mice, our study further delineates the functions of ID2 as the key transcription factor in follicle formation with the identification of its downstream effectors. Our result manifested the crucial role of BMP signaling pathway in primordial follicle formation. Meanwhile, we also for the first time demonstrated the differential regulations of ID proteins in oocytes during the process. Until now, we still do not know whether the redundancy exists for ID proteins during follicle formation. Future studies with multi‐genetic modified mouse models will be helpful to demonstrate their regulatory mechanisms in folliculogenesis.

Although multiple mechanisms have been described as causative factors in POI, in most cases, POI occurs because of the premature depletion of the primordial follicle pool (Rossetti et al., 2017). POI is highly heterogeneous, both in phenotype and etiology. As a complex multifactorial disease, genetic causes have been reported to link with 20%–25% of POI cases which is characterized by great genetic heterogeneity and may involve multiple genetic variants (Qin et al., 2015). To date, relatively few genes in human have been proven to cause POI by functional validation with animal models, including transcriptional regulators *Taf4b*, *Nobox*, *Figla*, and *Sohlh1*/*2* (Qin et al., 2015). A common phenotype of these mutant mice is the dramatic attrition of perinatal oocytes, resulting in POI with a much‐reduced ovarian reserve. In the study, when we tried to inspect the reported POI‐associated genes in our database, many genes were enriched as DEGs at stage transitions (20/69, 29%) (França & Mendonca, 2020). Due to the limitation of sample size, it is difficult to find specific novel genes that are implicated in the pathogenesis of POI by whole genome approaches. Additionally, for some candidates which knockout mice showed ovarian failure, no variants have been found in the corresponding human orthologous. Accordingly, our data will provide a good resource to screen POI candidate genes when combined with population‐based cohort studies. With the help of murine genetic models, the genetic basis of POI will be well delineated. It is important not only to understand ovarian physiology and pathology but also to provide genetic counseling and fertility guidance in the clinic.

In conclusion, during the process of cyst breakdown and follicle assembly, germ cells are orchestrated under tight transcriptional control toward the development of functional oocytes. In addition to the description of a set of TFs and their regulatory networks, our study further elucidated the important roles of ID1 and ID2 in primordial follicle formation and their different regulatory mechanisms. Further research will focus on finding key genes and their molecular mechanisms during follicle formation by using genetic animal models. This research will be helpful in delineating the genetic basis for primordial follicle reserve and in identifying the causative genes or genes responsible for POI.

## 4 EXPERIMENTAL PROCEDURES

### 4.1 Animal care and use

*Id2* ^flox/flox^ and *Id3* ^flox/flox^ transgenic mice were gifted from Prof. Yuan Zhuang at Duke University. *Vasa*‐Cre^/+^ mice were used to breed with *Id2* ^loxp/loxp^ or *Id3* ^flox/flox^ homozygotes to obtain *Vasa*‐Cre^/+^; *Id2* ^loxp/+^ or *Vasa*‐*Cre* ^/+^; *Id3* ^loxp/+^ female progeny, then the females were bred to *Id2* ^loxp/loxp^ or *Id3* ^loxp/loxp^ homozygous males to obtain *Id2* ^−/−^ or *Id3* ^−/−^conventional knockout mice (Zhang et al., 2014). All mice have a C57BL/6J genetic background. *Vasa*‐Cre mice were purchased from Model Animal Research Center of Nanjing University. Adult C57BL/6J female pregnant mice were obtained from Vital River Laboratories and housed in the animal facility at Nanjing Medical University. All animal protocols were approved by the Committee on the Ethics of Animal Experiments of Nanjing Medical University (IACUC1601220, 2101024). Mice were maintained under a 12/12‐hour dark‐light cycle at 22°C with free access to food and water.

### 4.2 Isolation of germ cells from newborn mouse ovaries

The newborn female pups were collected immediately after delivery and ovaries were harvested by carefully removing oviducts and ovarian bursa in calcium‐ and magnesium‐free Hanks balanced salt solution (HBSS). The ovaries were further digested in 500 µl HBSS supplemented with 0.25% trypsin, 1 mM ethylenediaminetetracetic acid (EDTA), and 0.01% DNase I and incubated at 37°C for 10 min with gentle agitation. To stop the digestion, 500 µl HBSS (plus 10% FBS) was added and the cell suspensions were centrifuged at 400 g for 5 min at 4°C. After aspirating the supernatant completely, the cells were resuspended in 500 µl HBSS. The dissociated single‐cell suspensions were transferred under the microscope (Nikon, SMZ1000), and single germ cell samples were picked up and transferred into the lysis buffer by mouth pipette.

The bioinformatics analysis protocols of the single‐cell sequencing data and other experimental procedures are detailed in Supplementary Experimental Procedures.

## CONFLICT OF INTEREST

The authors declare no conflict of interest.

## AUTHOR CONTRIBUTIONS

J.L., W.J.S, J.H.S., and Z.B.H. conceptualized the study. J.L. and Y.L.H. led the experimental design and development of the protocol with input from all authors. Q.Z.C. and J.C.D. provided bioinformatics analysis. Y.L.H., C.Z., X.D.W., J.Z.L., J.Z., Y.X., and X.W.D. performed the experiments. Y.Q.C. provided research tools and performed the experiments. W.J.S, J.L., Y.L.H., J.C.D., and Q.Z.C. designed the research and wrote the manuscript. J.H.S. and Z.B.H. corrected the manuscript. All authors analyzed and interpreted the data.

## Supporting information

Fig S1

Fig S2

Fig S3

Fig S4

Supplementary Material

Supplementary Material

## Data Availability

The raw single‐cell RNA sequencing data reported in this paper have been deposited in NCBI’s Gene Expression Omnibus (GEO) under the accession number GSE152407.
