# Supplementary figures and images for: Single‐cell RNA‐Seq reveals a highly coordinated transcriptional program in mouse germ cells during primordial follicle formation
Source: Aging Cell. 2021 Jun 26;20(7):e13424. doi: 10.1111/acel.13424 (PMC8282241; doi:10.1111/acel.13424)

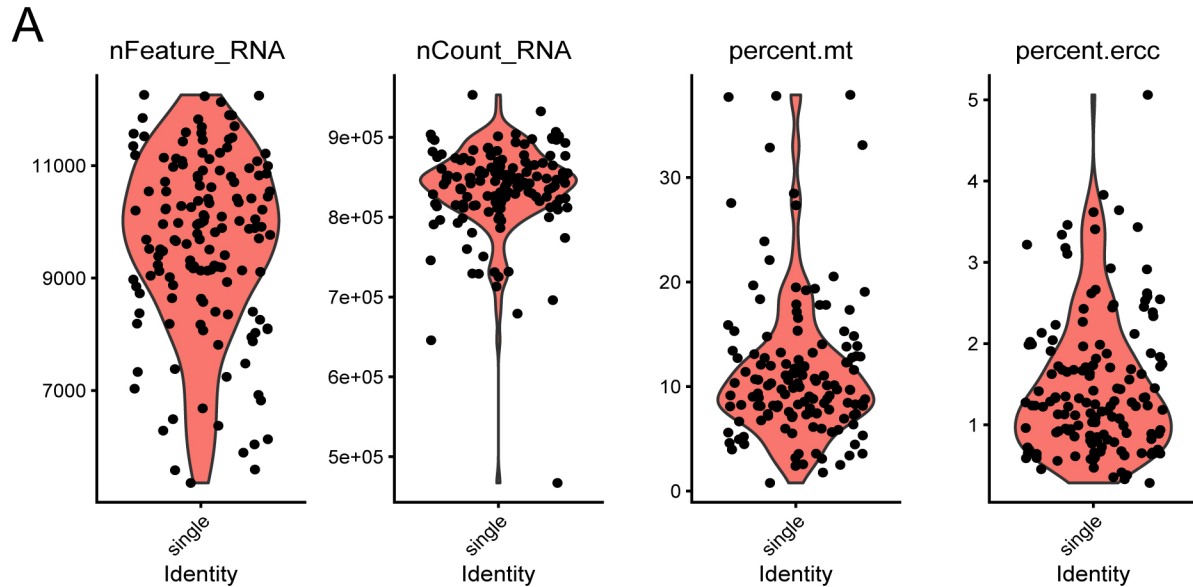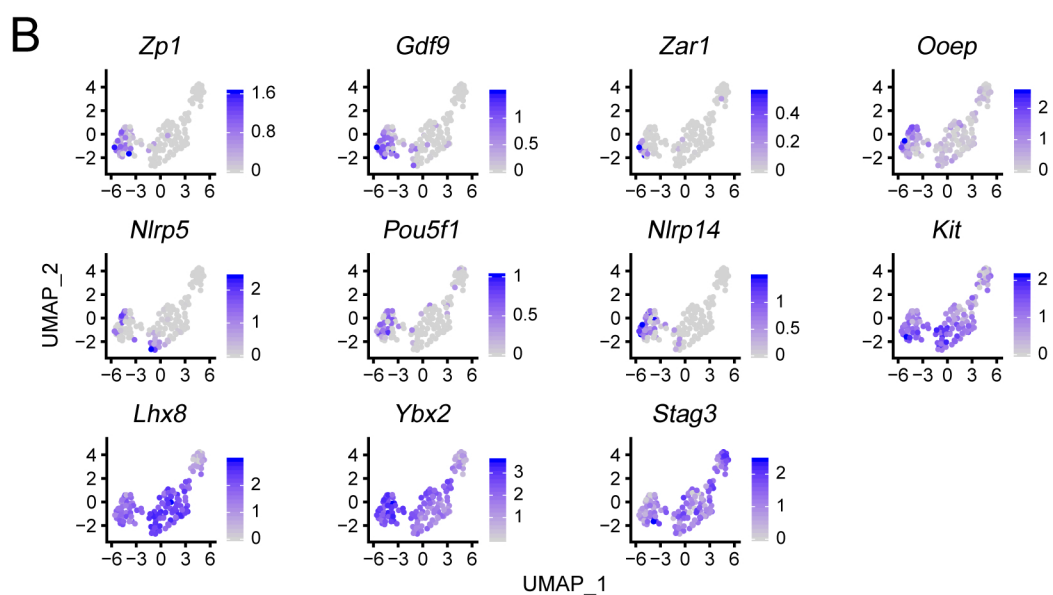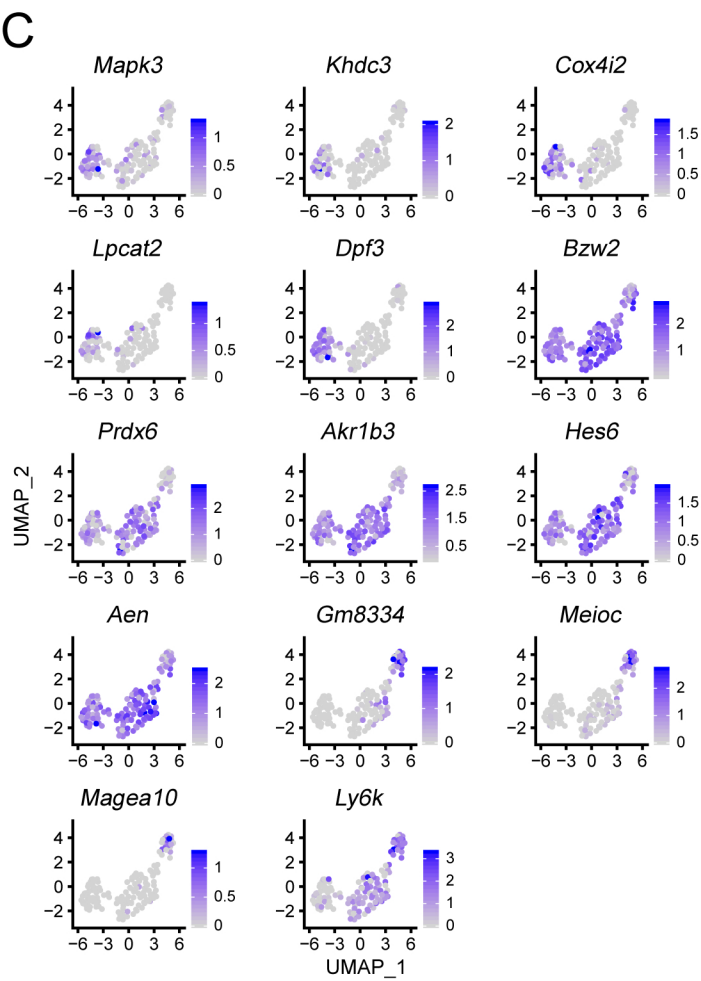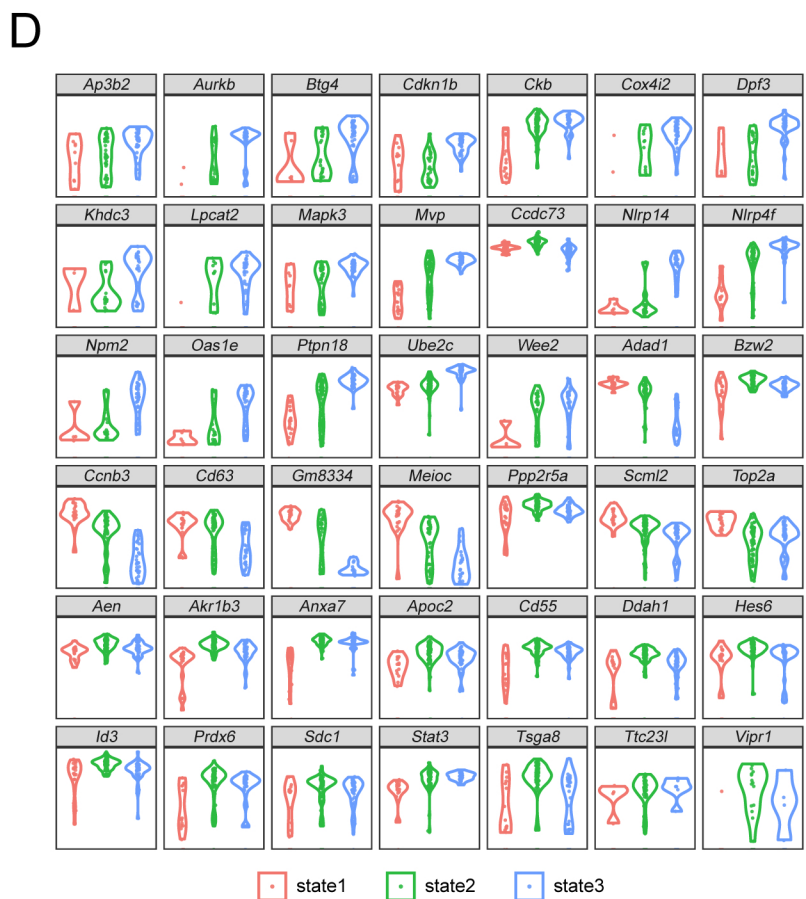

Supplement: Supplementary file 1 — Fig S1 [file ACEL-20-e13424-s005.pdf]

**A**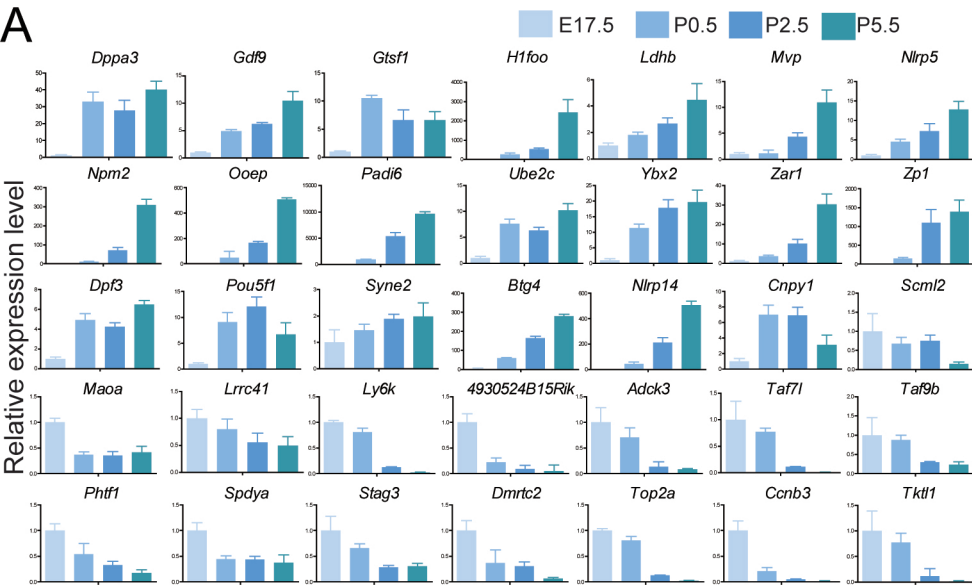**B**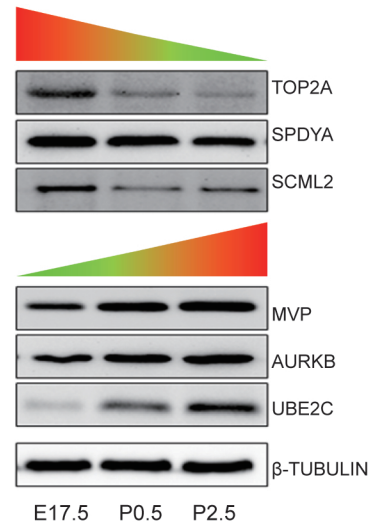

Supplement: Supplementary file 2 — Fig S2 [file ACEL-20-e13424-s001.pdf]

A

● state1 ● state2 ● state3

Expression

Pseudo-time

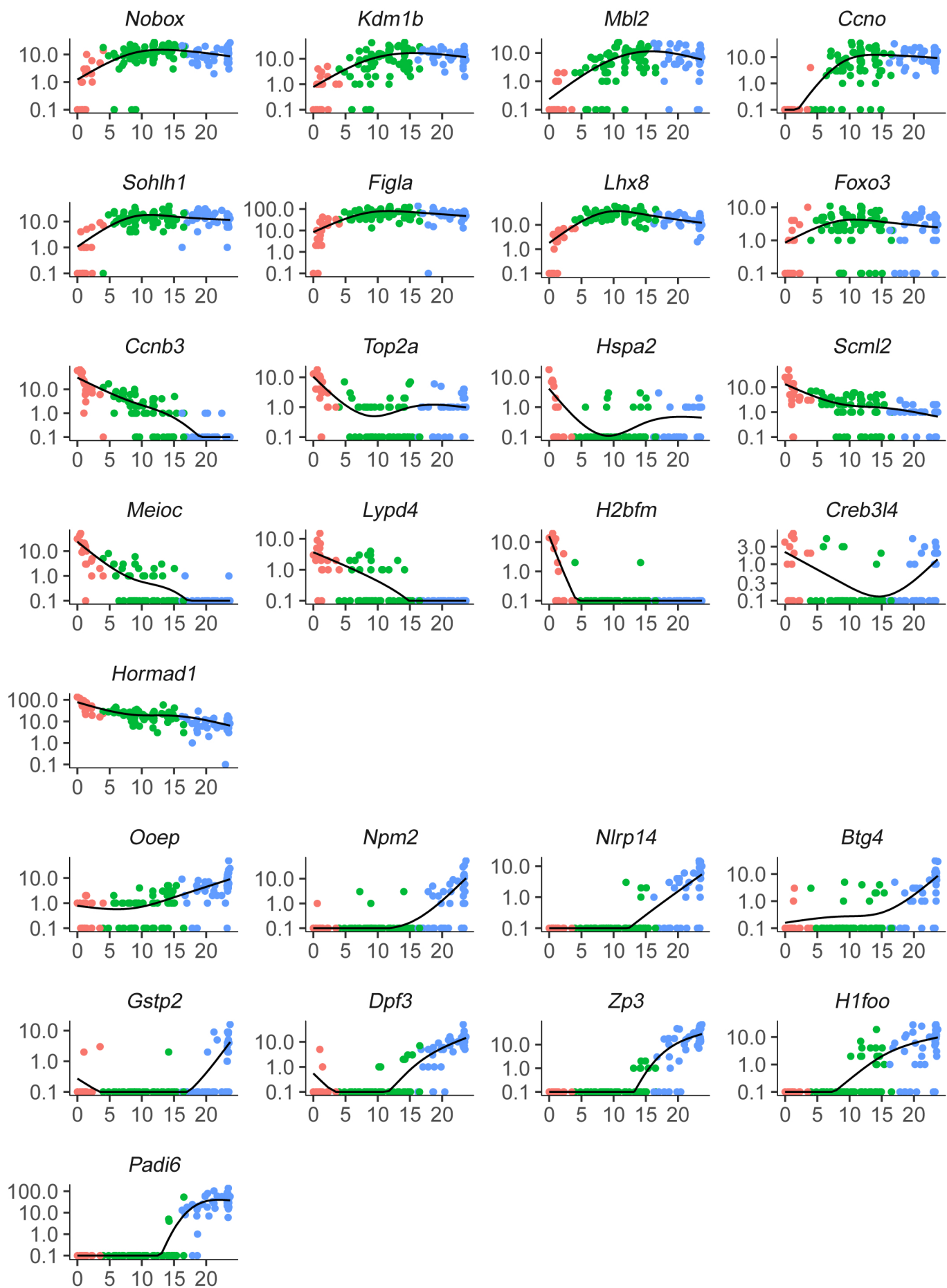

Supplement: Supplementary file 3 — Fig S3 [file ACEL-20-e13424-s006.pdf]

A

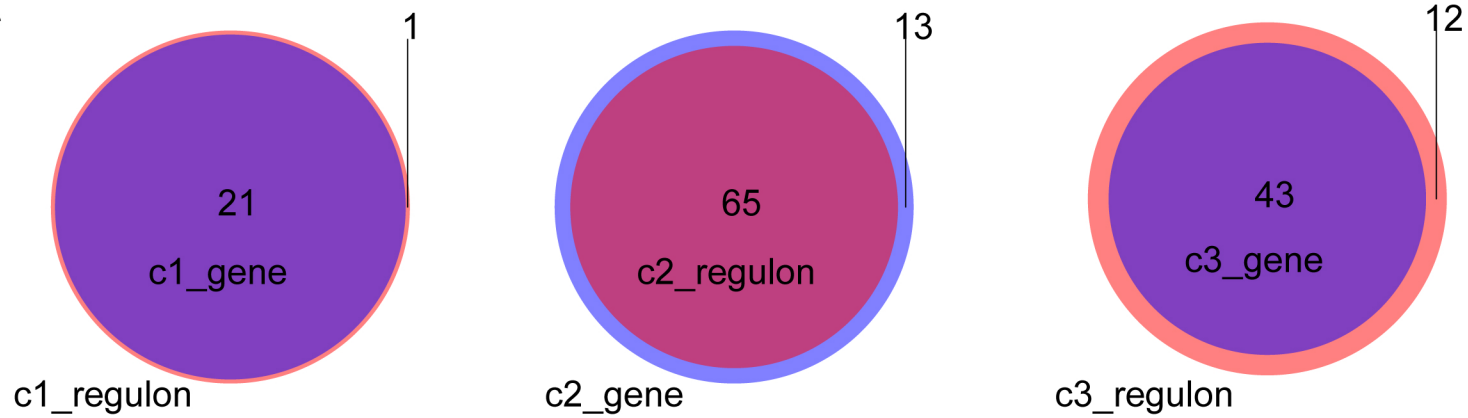

B

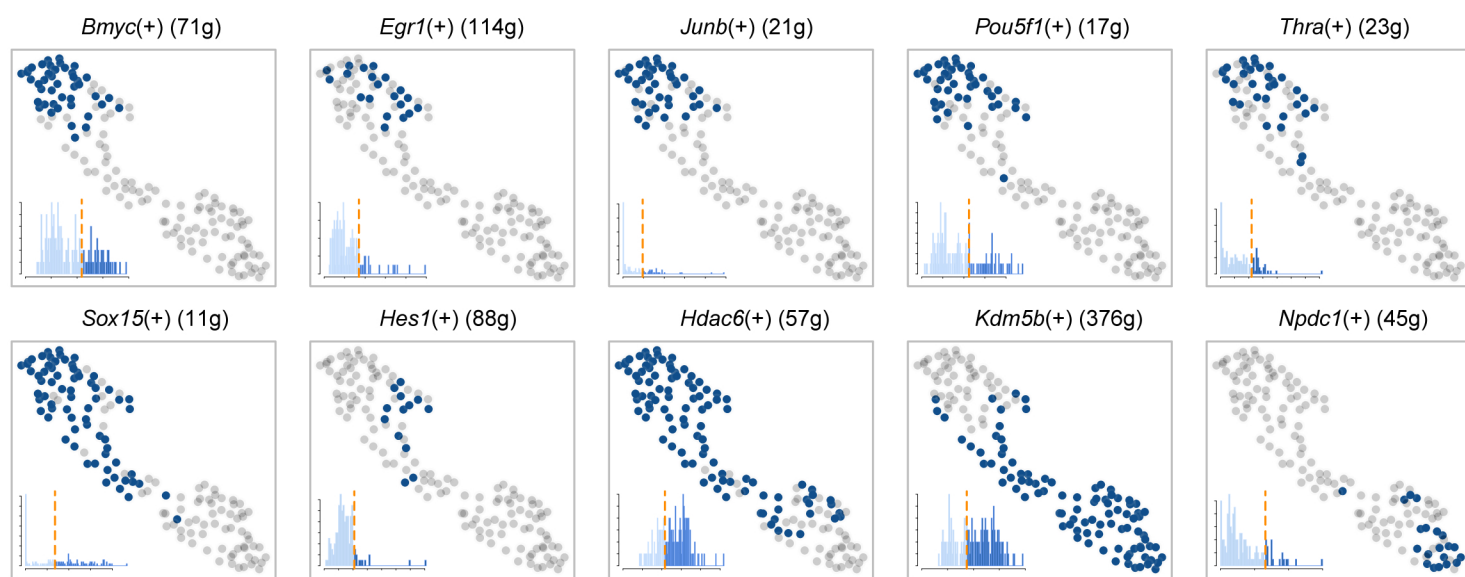

C

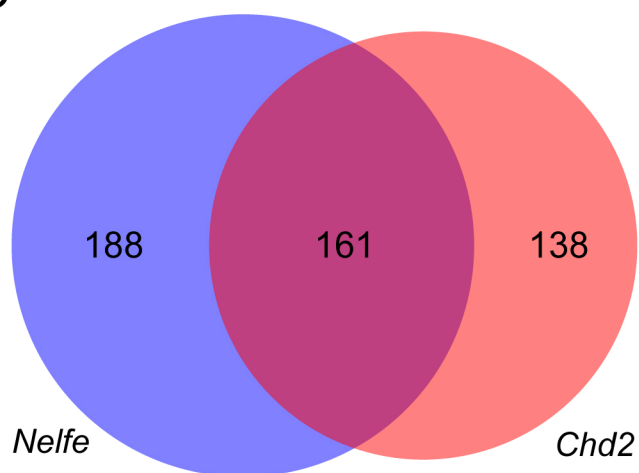

D

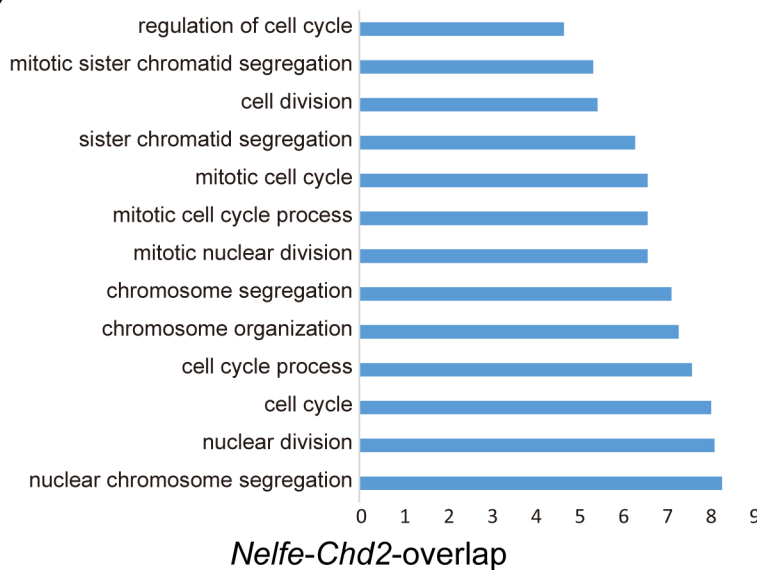

E

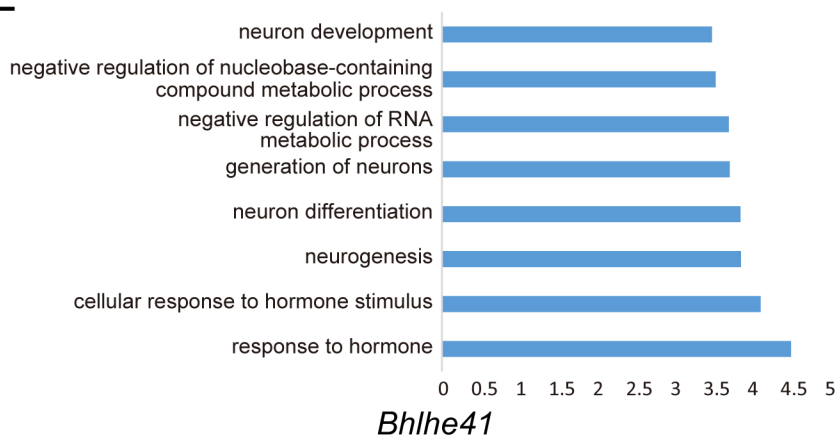

Supplement: Supplementary file 4 — Fig S4 [file ACEL-20-e13424-s004.pdf]
