# Supplementary material for: Single‐cell RNA‐Seq reveals a highly coordinated transcriptional program in mouse germ cells during primordial follicle formation
Source: Aging Cell. 2021 Jun 26;20(7):e13424. doi: 10.1111/acel.13424 (PMC8282241; doi:10.1111/acel.13424)
Supplement: Supplementary file 5 — Supplementary Material [file ACEL-20-e13424-s003.docx]

**Supplementary Experimental Procedures**

**Single-cell RNA-seq library preparation and sequencing**

Single cells were taken out from -80℃ freezer, and thawed on ice, 1μl of 10μM oligo-dT primer, 1μl of 10 mM dNTP mix and 0.1μl of ERCC spike-in RNA (1:100,000 dilution, Life Technologies, 4456740) were added to the lysis buffer. cDNA synthesis and amplification were performed following the Smart-seq2 protocol. After amplification, cDNA libraries were screened via quantitative PCR (1:10 dilution) for the expression of oocyte and pregranulosa cell marker genes (*Vasa*, *Foxl2*) and all *Foxl2* positive samples were removed from the sample pools. The left cDNA libraries were then checked on a Bioanalyzer instrument (Agilent) and the passed samples were finally chosen for sequencing. Sequence libraries were constructed by Annoroad Gene Technology Corporation (Beijing, China), and were sequenced by Illumina HiSeq 2500 for 125-bp paired-end sequencing with at least 20 million reads per single cell sample. During analysis, sequencing reads mapping to ERCC ‘spike-ins’ were used for estimation of technical ‘noise’ levels and for ‘calling’ of significantly highly variable genes by a published method (Brennecke et al.).

**Processing and quality control of single-cell RNA-seq raw data**

The quality control of all the single cell RNA-seq data was first checked by FastQC (v0.11.8) (Brown et al., 2017){Brown, 2017 #3}. We added the sequence of spike-in to the reference genome sequence (mm10) as the final reference genome sequence. The reads were mapped to the final reference genome sequence and quantified using Salmon (v0.10.0) (Patro et al., 2017) with seqBias parameter. Then, we used the Seurat (v3.1.2) (Satija et al., 2015) R package to perform calculation of the number of unique genes detected in each cell (nFeature_RNA), the total number of molecules detected within a cell (nCount_RNA), and the percentage of reads that map to the mitochondrial genome and ERCC spike-in (percent.mt, percent.ercc). Only the cells with nCount_RNA >500,000, percent.mt <35 and percent.ercc <4 were retained. Coding genes were quality-filtered by TPM (transcripts per million) greater than 0.1 in more than 10 cells. After filtering, 142 cells out of 146 cells with 12,103 coding genes were used for downstream analysis.

**Identification of cell types, cell trajectory and differential expressed genes (DEGs)**

The Seurat R package was used for this part of analysis. First, the TPM matrix of filtered cells and genes was constructed as the input for Seurat. To normalize the matrix, we applied function NormalizeData in Seurat with default parameters. Next, we scaled the data of all genes and selected 20 prior marker genes as the features to perform Principal Components Analysis (PCA). Then the JackStraw and RunUMAP analysis were used to evaluate principal components and reduced the dimensions, respectively. Finally, three clusters were identified with 21 cells in cluster 1, 78 cells in cluster 2, and 43 cells in cluster 3.

The DEGs between any two clusters were identified by function differentialGeneTest in Monocle2 package (v2.12.0) (Trapnell et al., 2014) with q-value < 0.001. Ultimately, there were 212 DEGs between cluster 1 and cluster 2, 925 DEGs between cluster 2 and cluster 3, and 1,174 DEGs between cluster 1 and cluster 3. GO analysis of DEGs was executed by Toppgene with p-value < 0.05.

Monocle2 (v2.12.0) (Trapnell et al., 2014) was also used to analyze pseudotime of single cells and ordered cells along a trajectory. The input of Monocle2 was the TPM matrix and the cells were annotated with the cluster numbers identified by Seurat. Next we estimated size factors and dispersions of our data. Pseudotime-dependent genes were identified with differentialGeneTest function in Monocle 2 with p-value < 0.05. Pseudotime dependent genes were divided into four expression patterns and Gene Ontology (GO) analysis of each patterns was executed by Toppgene (Chen et al., 2009) with p-value < 0.05. Then we reduced the data to two dimensions by DDRTree algorithm. The cells were ordered along the trajectory with reverse parameter that assigned the beginning.

**Transcriptional regulatory network analysis**

To identify the potential key transcriptional regulators in follicle formation, we used pySCENIC (v0.10.) (Aibar et al., 2017) to explore the potential transcriptional regulators. The 1,156 DEGs among three clusters with TPM matrix were taken as input. Based on the matrix, we constructed a co-expression modules between TF and potential target genes filtered by importance > 0.001. In each module, the potential targets which motif enrichment was significant to corresponding TF were regard as direct target genes. TF and all its direct target genes were defined as a regulon. We got 18 positive regulons after filtering through number of direct targets more than 10. All regulons were eventually scored by the active value using 20% of the ranked genes. We again identified and clustered cell clusters using active matrix of regulons as input by Seurat. Cells of each regulons were judged to be active according to the threshold determined by the distribution.

**Real-time PCR and high-throughput on-chip quantitative real-time PCR analyses**

Ovaries were collected at E17.5, P0.5, P2.5 and P5.5 and total RNA was isolated by RNAprep Pure Micro Kit (DP420, TIANGEN, Beijing, China) according to the manufacturer’s instruction. After reverse-transcription, Eva Green-based qPCR was performed using SsoFast EvaGreen Supermix With Low ROX (Bio-Rad Laboratories, PN 172-5211) on an ABI StepOnePlus platform (Thermo Fisher Scientific, Waltham, USA). The ΔΔCt method was used to quantify various mRNAs with the actin amplification signal as the internal control. The specificity of the PCR products was assessed by melting curve analyses and amplicon size was determined by electrophoresis in 2% Agarose gels.

For high-throughput qPCR profiling, a total of 18 oocytes samples with each sample containing 30 oocytes were collected from E17.5 (6 samples), P0.5 (6 samples) and P2.5 (6 samples) ovaries respectively by the method mentioned above. The cDNA synthesis and amplification were performed in the same way as it was performed for RNA-seq according to the Smart-seq2 protocol. And gene expression levels for the 48 selected genes in oocyte samples were measured with qPCR on a 48.48 Dynamic Array IFC using the Fluidigm Biomark HD system. We used a conservative Ct of 30 as LOD. Gene expression was defined on a log2 scale as: log2 expression=LOD-Ct, as described in the Fluidigm manual (PN 100-5066 E3). The raw data of the qPCR were in Supplementary Tables S3. All Primer sequences in both qPCR analyses were listed in Supplementary Table S6.

**RNA fluorescent in situ hybridization**

Ovaries at E17.5, P0.5 and P5.5 were collected and fixed in 4% paraformaldehyde at 4°C for at least 8 h. Following with consecutive dehydration in 10% sucrose and 30% sucrose, the tissues were embedded in OCT cryopreservation solution (NEG 50, Thermo Fisher Scientific) for frozen sections. For prob preparation, total RNAs from newborn ovaries (P0.5) were isolated for cDNA syntheses. After PCR with specific primers, the amplified fragments were cloned into the pGEM-T vector for the synthesis of antisense and sense probes from T7 or SP6 [promoter](https://www.sciencedirect.com/topics/biochemistry-genetics-and-molecular-biology/promoter-genetics) by using the fluorescein RNA Labelling Kit (Roche, Cat# 11685619910). All primers for probe preparation were listed in Supplementary Table S9. Fluorescent RNA in situ hybridization (FISH) was performed according to the method described previously (Ishii et al., 2004). After washing and blocking, the hybridized sections were incubated with anti-fluorescein-POD (Roche, Cat# 11426346910) at RT for 1 h. The sections were then sequentially incubated with TSA-488/594 (Thermo Fisher Scientific, Cat# [B40953](https://www.thermofisher.com/order/catalog/product/B40953)/ [B40957](https://www.thermofisher.com/order/catalog/product/B40957)) for 10 min. Nucleus was stained with DAPI and slides were mounted with the Gold anti-fade reagent (Molecular Probes).

**Immunohistochemistry**

Newborn ovaries (P0.5) were fixed in 10% buffered formalin for paraffin embedding and sectioning. After deparaffinization and rehydration, endogenous peroxidase activity was blocked by incubation in 3% hydrogen peroxide in methanol for 20 min. Antigen retrieval pretreatment was carried out by boiling the sections in 0.01M citrate buffer, PH6.0 for 10min. Immunohistochemistry analyses were performed using a SPlink Detection Kits (Zhong Shan Jin Qiao) with specific antibodies overnight at 4°C. Negative controls were performed by incubation with non-immune IgGs.

**Immunoblotting**

Ovarian proteins were extracted by RIPA lysis buffer (Beyotime Institute of Biotechnology) with protease inhibitor cocktails (Amresco). Protein concentrations were measured by a BCA assay (Thermo Fisher Scientific). After separation by electrophoresis on a 10% SDS-PAGE gel, proteins were electronically transferred to polyvinylidene fluoride membranes (PVDF) (IPVH00010, Millipore). Membranes were blocked in 5% skimmed milk-TBST (TBS containing 0.1% Tween 20) for 30min and incubated overnight at 4°C with primary antibodies. After rinsing thoroughly with TBST (ZSGB-BIO, Beijing, China), the membranes were incubated for 1 h at room temperature with the appropriate secondary antibodies (ZSGB-BIO, Beijing, China). Finally, the membranes were visualized using the Super Signal chemiluminescent detection system (34080, Thermo).

**Immunofluorescence**

Frozen ovarian sections were washed in PBST (PBS containing 0.05% Triton X-100, pH 7.4) for 3 times. Then, after blocking with 5% bovine serum albumin (BSA, Sigma) for 30 min, slides were incubated with primary antibodies in 1% BSA at 4 °C overnight. After washing with PBST, the sample was incubated with Alexa Flour 488 or Alexa Flour 594-conjugated appropriate secondary antibodies diluted with PBS at RT for 1 h. Next, the slides were washed in PBS, and nuclei were stained with 0.01mg/ml Hoechst 33342 (H1399, Invitrogen) for 20min. Images were taken under a laser scanning confocal microscope (LSM 700, Zeiss, German).

**Oocyte and Follicle Counting**

To assess follicular formation in *Id2* knockout mice, P2.5 ovaries were collected for paraffin embedded-sections. We use P27 staining to differentiate germ cells during follicle assembly: germ cells negative for P27 staining and without surrounding of P27 positive somatic cells are germ cells in cyst; germ cells negative for P27 staining but with surrounding of several P27 positive somatic cells are germ cells undergoing CBD and germ cells with P27 positive staining in nuclei are oocytes in primordial follicles (Rajareddy et al., 2007). Germ cells at different developmental states were then counted as previously reported (Zhang et al., 2017). Briefly, two serial sections from the largest cross-section through the center of each ovary were chosen for follicle/oocyte counts, and the average was used as the follicle/oocyte number of one ovary. Follicle counting on adult ovaries (4 W and 8 M) was performed on serial sections (5 µm) after H&E staining. Follicles at different developmental stages were counted every fifth section according to the fractionator and nucleator principles(Sun et al., 2015). All follicle counts were repeated in the ovaries from at least three independent experiments (n=5-6 ovaries/group).

**Statistical Analysis**

All measurements were performed independently at least in triplicate. For real-time RT-PCR and follicle counting, the data were expressed as mean + SD and one-way ANOVA were used to evaluate differences between groups. A value of *P*<0.05 or *P*<0.01 was considered to be statistically significant.

**Antibodies**

All antibodies used in this study were listed in Supplementary Tables S7.

**Reference**

Aibar, S., González-Blas, C.B., Moerman, T., Huynh-Thu, V.A., Imrichova, H., Hulselmans, G., Rambow, F., Marine, J.-C., Geurts, P., Aerts, J.*, et al.* (2017). SCENIC: single-cell regulatory network inference and clustering. Nature methods *14*, 1083-1086.

Brennecke, P., Anders, S., Kim, J.K., Kolodziejczyk, A.A., Zhang, X., Proserpio, V., Baying, B., Benes, V., Teichmann, S.A., Marioni, J.C.*, et al.* (2013). Accounting for technical noise in single-cell RNA-seq experiments. Nature methods *10*, 1093-1095.

Brown, J., Pirrung, M., McCue, L.A., and Wren, J. (2017). FQC Dashboard: integrates FastQC results into a web-based, interactive, and extensible FASTQ quality control tool. Bioinformatics *33*, 3137-3139.

Chen, J., Bardes, E.E., Aronow, B.J., and Jegga, A.G. (2009). ToppGene Suite for gene list enrichment analysis and candidate gene prioritization. Nucleic Acids Research *37*, W305-W311.

Ishii, T., Omura, M., and Mombaerts, P. (2004). Protocols for two- and three-color fluorescent RNA in situ hybridization of the main and accessory olfactory epithelia in mouse. J Neurocytol *33*, 657-669.

Patro, R., Duggal, G., Love, M.I., Irizarry, R.A., and Kingsford, C. (2017). Salmon provides fast and bias-aware quantification of transcript expression. Nature methods *14*, 417-419.

Rajareddy, S., Reddy, P., Du, C., Liu, L., Jagarlamudi, K., Tang, W., Shen, Y., Berthet, C., Peng, S.L., Kaldis, P.*, et al.* (2007). p27kip1 (cyclin-dependent kinase inhibitor 1B) controls ovarian development by suppressing follicle endowment and activation and promoting follicle atresia in mice. Mol Endocrinol *21*, 2189-2202.

Satija, R., Farrell, J.A., Gennert, D., Schier, A.F., and Regev, A. (2015). Spatial reconstruction of single-cell gene expression data. Nat Biotechnol *33*, 495-502.

Sun, X., Su, Y., He, Y., Zhang, J., Liu, W., Zhang, H., Hou, Z., Liu, J., and Li, J. (2015). New strategy for in vitro activation of primordial follicles with mTOR and PI3K stimulators. Cell cycle (Georgetown, Tex) *14*, 721-731.

Trapnell, C., Cacchiarelli, D., Grimsby, J., Pokharel, P., Li, S., Morse, M., Lennon, N.J., Livak, K.J., Mikkelsen, T.S., and Rinn, J.L. (2014). The dynamics and regulators of cell fate decisions are revealed by pseudotemporal ordering of single cells. Nat Biotechnol *32*, 381-386.

Zhang, J., Liu, W., Sun, X., Kong, F., Zhu, Y., Lei, Y., Su, Y., Su, Y., and Li, J. (2017). Inhibition of mTOR Signaling Pathway Delays Follicle Formation in Mice. J Cell Physiol *232*, 585-595.
