# Supplementary material for: Single‐cell RNA‐Seq reveals a highly coordinated transcriptional program in mouse germ cells during primordial follicle formation
Source: Aging Cell. 2021 Jun 26;20(7):e13424. doi: 10.1111/acel.13424 (PMC8282241; doi:10.1111/acel.13424)
Supplement: Supplementary file 6 — Supplementary Material [file ACEL-20-e13424-s002.docx]

**Supplementary Figures and Figure Legend**

**Figure S1.** Quality control and gene expressions in clusters. **(A)** Violin plots of the number of unique genes detected in each cell (nFeature_RNA), the total number of molecules detected within a cell (nCount_RNA), and the percentage of reads that map to the mitochondrial genome and ERCC spike-in (percent.mt, percent.ercc). **(B)** Distributions of feature genes in clusters. The feature genes refer to genes selected to perform PCA analysis during cell clustering. **(C)** Identified DEGs with specific distributions in three clusters. **(D)** Volcano Plots of DEGs in three states.

**Figure S2**. Expressions of representative DEGs in ovaries at different developmental stages. Ovaries were collected at E17.5, P0.5, P2.5 and P5.5, respectively. **(A)** RT-PCR of representative DEGs. The expression of all tested mRNAs was normalized to the E17.5 samples. **(B)** Western Blot of representative DEGs. Ovaries were collected at E17.5, P0.5 and P2.5, respectively. β-TUBULIN was used as the internal control. All data were presented as mean ± SD of at least three repeats.

**Figure S3.** Marker gene expression trends along the pseudotime line. **(A)** Expressions of selected marker genes along the pseudo-time line.

**Figure S4.** Analysis based on the regulon active score and GO of regulons. **(A)** The Venn diagram shows the overlap between cell clusters based on gene expression and regulon active scores. **(B)** Binary active distribution of regulons. The histograms show the distribution of regulon active scores. **(C)** The Venn diagram showing the overlap between *Chd2* and *Nelfe* regulons. **(D)** GO terms in overlapped target genes between *Chd2* and *Nelfe* regulons together with the enrichment significance scores. **(E)** GO terms in *Bhlhe41* regulons together with the enrichment significance scores.

**Figure S5.** The diagram of dynamic gene expression and regulation during follicle assembly. The uniform developmental trace of single cells along the pseudo-timeline demonstrates the transition from germ cells to a functional oocyte. STI, stage transition I, from cyst to cyst breakdown (CBD) stage; STII, stage transition II, from cyst to follicle stage. The stage transitions are accompanied with dynamic gene expressions and under the transcriptional regulations. The boxes under STI and STII show the stage-specific transcriptional regulons. The trends of pseudo-dependent genes along the developmental trajectory are plotted with representative GO terms and marker genes.

**Supplementary Table Legend**

**Table S1.** The mapping ratio and cluster based on gene expression of cells.

**Table S2.** DEGs identified between two cluster and GO terms enrichment analysis of DEGs.

**Table S3.** High-throughput real-time PCR by low cells

**Table S4.** Pseudotime-dependent genes and GO of pseudotime-dependent genes.

**Table S5.** Transcription factors with corresponding target genes; Cells cluster based on regulon active scores; GO terms enrichment analysis of *Bhlhe41* regulons; GO terms enrichment analysis of overlap genes between *Nelfe* and *Chd2* regulons.

**Table S6.** Primers sequence.

**Table S7.** Antibodies
